# The *Saccharomyces cerevisiae* Yta7 ATPase hexamer contains a unique bromodomain tier that functions in nucleosome disassembly

**DOI:** 10.1016/j.jbc.2022.102852

**Published:** 2022-12-30

**Authors:** Feng Wang, Xiang Feng, Qing He, Hua Li, Huilin Li

**Affiliations:** Department of Structural Biology, Van Andel Institute, Grand Rapids, Michigan, USA

**Keywords:** nucleosome remodeler, AAA+-ATPase, Yta7, cryo-EM, histone disaggregase, BIM, BRD-interacting motif, BRD, bromodomain, CV, column volume, HD, helical subdomain, MBP, maltose-binding protein, N-tail, N-terminal peptide, PL, pore loop, Yta7, yeast Tat (transactivator of transcription)–binding analog 7

## Abstract

The *Saccharomyces cerevisiae* Yta7 is a chromatin remodeler harboring a histone-interacting bromodomain (BRD) and two AAA+ modules. It is not well understood how Yta7 recognizes the histone H3 tail to promote nucleosome disassembly for DNA replication or RNA transcription. By cryo-EM analysis, here we show that Yta7 assembles a three-tiered hexamer with a top BRD tier, a middle AAA1 tier, and a bottom AAA2 tier. Unexpectedly, the Yta7 BRD stabilizes a four-stranded β-helix, termed BRD-interacting motif (BIM), of the largely disordered N-terminal region. The BIM motif is unique to the baker’s yeast, and we show both BRD and BIM contribute to nucleosome recognition. We found that Yta7 binds both acetylated and nonacetylated H3 peptides but with a higher affinity for the unmodified peptide. This property is consistent with the absence of key residues of canonical BRDs involved in acetylated peptide recognition and the role of Yta7 in general nucleosome remodeling. Interestingly, the BRD tier exists in a spiral and a flat-ring form on top of the Yta7 AAA+ hexamer. The spiral is likely in a nucleosome-searching mode because the bottom BRD blocks the entry to the AAA+ chamber. The flat ring may be in a nucleosome disassembly state because the entry is unblocked and the H3 peptide has entered the AAA+ chamber and is stabilized by the AAA1 pore loops 1 and 2. Indeed, we show that the BRD tier is a flat ring when bound to the nucleosome. Overall, our study sheds light on the nucleosome disassembly by Yta7.

Nucleosomes are a formidable barrier to transcription and other DNA-dependent processes in eukaryotes (1, 2). A set of chromatin-related factors have evolved to disassemble nucleosomes to facilitate DNA replication, transcription, DNA damage detection, and repair (3, 4, 5, 6). The *Saccharomyces cerevisiae* Yta7 (yeast Tat [transactivator of transcription]-binding analog 7) is a chromatin-associated AAA+ (ATPases associated with various cellular activities) ATPase that maintains the balance of nucleosome density of the chromatin (7, 8, 9, 10, 11). Yta7 is type II ATPase containing two ATPase domains (AAA1 and AAA2) (12), which is conserved in eukaryotes (13) and expected to assemble a 910 kDa hexamer. The human homolog ATAD2 increases chromatin dynamics and gene transcription and is frequently overexpressed in various cancers (14). The *Saccharomyces pombe* homolog Abo1 has also been shown to act on nucleosome (15). Yta7, ATAD2, and Abo1 are the only AAA+ ATPase chaperone that acts specifically on histone to regulate gene transcription and DNA replication (8, 13).

Yta7 was recently found to be a nucleosome segregase that promotes nucleosome disassembly for chromatin replication in an S phase cyclin-dependent kinase–dependent manner (16) (Fig. 1*A*). Yta7 was also shown to disassemble the Cse4/H4 tetramer and hand over the unfold product to Scm3 for deposition to centromere (17). Yta7 contains a largely disordered N-terminal region (1–416 amino acid) that includes an acidic N-terminal region (118–185 amino acids), 13 phosphorylation sites that likely contribute to the H3 recognition (13, 18), and two tandem AAA+ domains (AAA1 and AAA2) that each contains an α/β subdomain and a helical subdomain (HD) (7, 15, 17) (Fig. 1*B*). The AAA1 is an active ATPase, whereas the AAA2 has no ATPase activity because of a lack of nucleotide-binding motif. Inserted in the AAA2 HD is a bromodomain (BRD) at the C terminus. The BRD is a conserved protein-interacting module that recognizes histones and regulates ATP-dependent chromatin remodeling (19, 20, 21). A typical BRD fold is 110-residue long and composed of a four-helix bundle that binds to acetylated peptides. The structures of several canonical BRDs have been determined (22), and several BRD-containing proteins are being targeted for anticancer drug development (21, 23, 24, 25, 26). Interestingly, the Yta7 BRD is extended and noncanonical and binds the H3 N-terminal peptide (N-tail) regardless of acetylation status (18).

**Figure 1:**
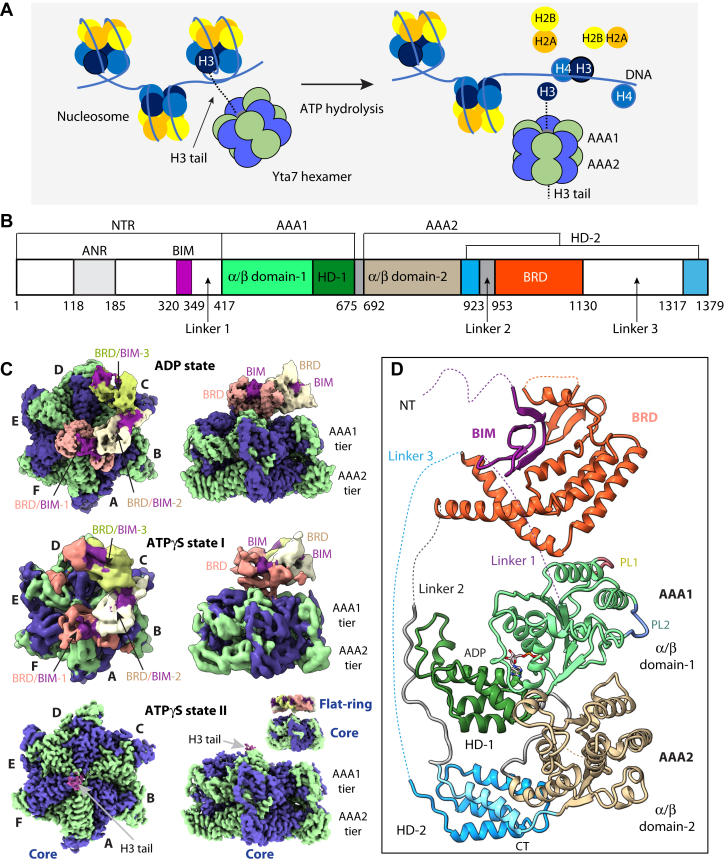
**Structures of the *Saccharomyces cerevisiae* Yta7 ATPase hexamer.***A*, a sketch showing that Yta7 unfolds histone H3 to remove nucleosome from chromatin. *B*, domain architecture of the yeast Yta7. Unresolved regions in the EM map are in *white*. *C*, *top and side views* of the EM maps of the Yta7 hexamer bound to ADP and ATPγS in states I and II. Alternating subunits are in *blue* and *green*. The three resolved BRDs are in *salmon*, *cream*, and *olive*, respectively, and the associated NTRs are in *magenta*, the H3 N-tail is in *plum*. The ATPγS-bound state II was reconstructed as a 3-Å AAA+ core region and as a lower resolution full complex that reveals a ring-shaped BRD–BIM tier (*inset* in the side view of the AAA core). *D*, structure of a Yta7 protomer with individual domains colored as in (*B*). BIM, BRD-interacting motif; BRD, bromodomain; N-tail, N-terminal peptide; NTR, N-terminal region.

The first structural insight was obtained by a recent structural study of the *S. pombe* homolog Abo1, revealing stacked AAA1 and AAA2 rings, with the rings adopting either an open spiral or a closed configuration depending on the nucleotides (15). However, the study used an N-terminal region truncated protein, did not resolve the BRD, and captured a translocating peptide of an unknown sequence. Therefore, the molecular mechanism underlying histone recognition and unfolding by Yta7 is not well understood. We have performed a structure–function analysis on the purified full-length Yta7. We found that the Yta7 BRD stabilizes a β-helix motif of the N-terminal region and that this motif promotes BRD oligomerization and contributes to the nucleosome recognition. Furthermore, we found that the BRD tier exists in both spiral and flat ring forms, and that nucleosome binding converts the spiral BRD tier to a flat ring to allow H3 peptide entry into the Yta7 AAA+ chamber. Our study provides insights into nucleosome recognition and unfolding mechanism of Yta7 and contribute to understanding of the nucleosome disassembly.

## Results

### The overall Yta7 structure

We purified the full-length Yta7 *via* a genetically inserted 3xFLAG tag (Fig. S1, *A* and *B* and Table S1). As expected, the purified protein hydrolyzed ATP and interacted with either a 24-residue H3 N-tail or a nucleosome (Fig. S1, *C* and *D*), and Yta7 bound to the H3 peptide with a similar affinity in the presence of ADP or ATPγS (Fig. S1*E*). We went on to first determine a cryo-EM map of the Yta7 in the presence of 2 mM ADP at an average resolution of 3.1 Å (Figs. 1, *C* and *D*, S2 and S3 and Table S2). As expected, Yta7 assembled a hexamer similar to the pombe homolog Abo1 (15). We resolved three of the six BRDs at a lower resolution of 3.9 Å (Fig. S2). The three unresolved BRD domains are expected to be a part of the spiral, but they are too flexible to be visualized in 3D reconstruction. The Yta7 structure is three tiered with a spiral BRD tier on top followed by the AAA1 and AAA2 tiers, has a dimension of 150  × 150 × 135 Å, and adopts an asymmetrical overall configuration. The active AAA1 coordinates an ADP between the α/β subdomain and the HD, and no ADP is present between the α/β subdomain and the HD in the inactive AAA2 (Fig. 1*D*). The six AAA2 domains form a flat and symmetric ring at the bottom tier. But the six AAA1 domains form a shallow spiral ring enabled by the six well-ordered but differently configured linkers between the AAA1 and AAA2 domains.

Interestingly, we found that the C-terminal BRD domain is inserted between the first and second α-helices of the AAA2 HD subdomain at the bottom. However, the BRD domain is positioned all the way on the top of the hexamer. The top BRD location is enabled by the presence of two long linkers—the 37-residue linker 2 and the 187-residue linker 3 (Fig. 1, *B* and *D*). These linkers are largely disordered except for the first half of linker 2 that is stabilized by the AAA1 HD. Such Yta7 architecture contrasts with the general protein disaggregase ClpB/Hsp104 in which an essential middle domain is inserted in the AAA1 tier and is located to the side of the hexamer (12, 27, 28, 29). Based on the density feature and an AlphaFold structural prediction (30), we unexpectedly identified a 30-residue motif in the N-terminal region (Gly320–Asn349) that interacted with the BRD and referred to it as a BRD-interacting motif (BIM) (Fig. 1, *C* and *D*). The details of the motif will be described later.

### The BRD tier exists in a spiral and a flat-ring state in the presence of ATPγS

To understand how Yta7 interacts with the H3 tail, we next performed cryo-EM on the Yta7–H3 N-tail (1–24 amino acids) complex in the presence of 2 mM ATPγS. We derived two 3D maps, one without H3 N-tail density at an estimated resolution of 5.6 Å and the other with a clear H3 N-tail density at 3.0 Å overall resolution (Figs. 1*C*, S4 and S5). The 5.6 Å ATPγS-bound Yta7 map resembled the ADP-bound Yta7 structure with three partially ordered BRD domains forming the right-handed spiral and was termed state I (Fig. S6*A*). Accordingly, the 3.0 Å map with bound H3-tail was termed state II. In state II, the top BRD tier is highly flexible and invisible in the high-resolution map. But further 3D classification and focused refinement revealed at a low resolution (9.7 Å) that the BRD tier is in a flat-ring configuration, in which the H3 peptide now could thread into the AAA+ chamber from the center of the ring (Figs. 1*C* and S6*B*). Therefore, we suggest that the flexible Yta7 BRD tier switches between a spiral and a flat ring in the presence of ATPγS.

### Both BRD and BIM contribute to histone H3 binding

**Figure 2:**
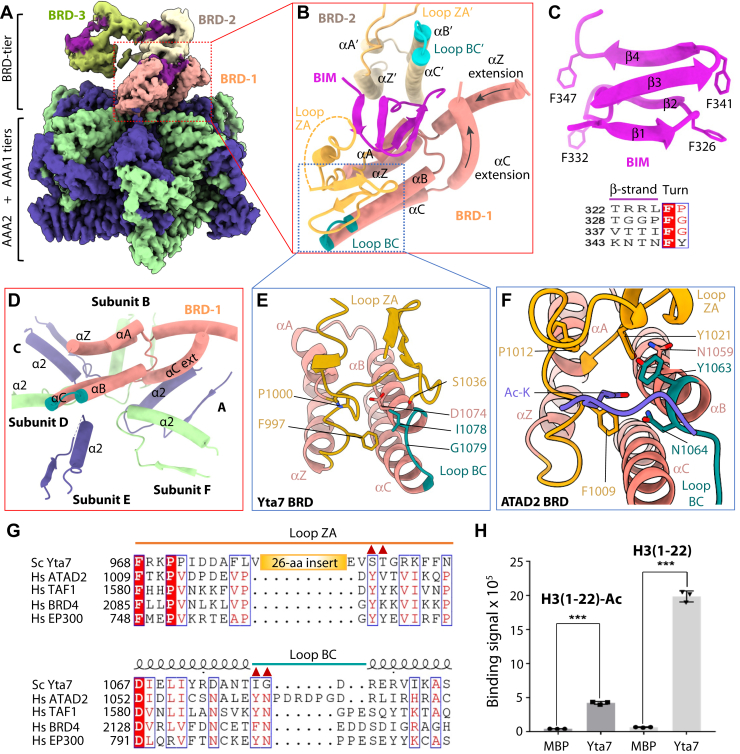
**Structure of the Yta7 bromodomain (BRD) and the novel BRD-interacting motif (BIM).***A*, composite EM map of the ADP-bound Yta7 hexamer colored in the same scheme as for Figure 1*D*. The EM densities of the second (*in cream*) and third BRD (*in olive*) and their associated BIMs (*in magenta*) were derived separately and displayed at a lower threshold. *B*, interface of BRD–BIM-1 and BRD–BIM-2. The conserved α-helices (αA–αC, αZ) and their connecting loops (ZA in *yellow* and BC in *teal*) of the BRD fold are labeled. *C*, structure of the BIM motif in *cartoon*. *Lower panel* shows the conserved Phe at each turn of the β-helices. *D,* interface of BRD-1 and the top region of AAA1 hexamer. The *top* α2 helix of each subunit and the conserved α-helices (αA–αC, αZ) of the BRD-1 are labeled. *E* and *F*, comparison of the substrate-binding pocket of BRD in Yta7 (*D*) and in ATAD2 (*E*; Protein Data Bank ID: 4QUT). Key residues involved in H3 N-tail binding are labeled. *G*, sequence alignment of the ZA and BC loops of five BRDs. The *red triangles* mark the conserved residues that bind to the acetylated lysine in ATAD2 and PCAF; these residues are not conserved in Yta7. Hs, *Homo sapiens*; Sc, *Saccharomyces cerevisiae. H*, interaction between Yta7 and unmodified and acetylated H3 N-tails (1–22 amino acids). All values represent means ± SD obtained from three independent experiments performed in triplicate. ∗∗∗ significantly different from control (*p* ≤ 0.001, one-tailed *t* test). N-tail, N-terminal peptide.

As described previously, we have observed the BRD tier in a spiral configuration in both ADP-bound state and ATPγS-bound state I. Because the ADP-bound state has better defined BRD region, we hereby describe the structure of the ADP-bound state as a surrogate to the ATPγS-bound state I. The spiral rises above the AAA1 tier with the proximal BRD–BIM blocking the peptide entry into the AAA+ chamber (Figs. 1*C*, 2, *A*–*D* and S7). The BRD domain spans from Pro954 to Leu1136 and contains the conserved core of the four-helix bundle (αZ, αA–αC) (Fig. 2*B*). However, the core is expanded by one α-helix preceding the αZ (αZ extension) and another α-helix following αC (αC extension). The expansion is unique to Yta7 and ATAD2 and is absent in a canonical BRD. The BIM is cradled in the U-shaped BRD burying a surface of 890 Å^2^, over 30% of the total surface. The BIM has a right-handed 2-turn β-helix fold, a fold frequently involved in protein–protein interaction (Fig. 2, *B* and *C*). Interestingly, at each turn of the four β-strands is located a hydrophobic phenylalanine residue. In Yta7, the BRDs of different subunits have no direct contact with each other, and it is the BIM that mediates and cements the intersubunit BRD–BRD interaction. However, the BIM is not conserved and appears to be unique to the *S. cerevisiae* Yta7 (Fig. S8). The bottom (proximal) BRD is located above the α2 helices of the AAA1 tier (Fig. 2*D*). Specifically, the BRD αB helix interacts with α2 helix of subunits E and F, the αC contacts the pore loop (PL) of subunits C and D, and the extend αC helix interacts with α2 helix of subunit A. Therefore, the BIM-stabilized BRD tier is held on top of the hexamer by weak and nonspecific interactions with the AAA1 tier.

In the canonical BRDs, the ZA and BC loops form a pocket that specifically recognize acetylated histone peptides (19, 22). The ZA loop of Yta7 BRD has a 26-residue insertion compared with canonical BRDs, and the loop is stabilized by the BIM (Fig. 2, *E*–*G*). The longer ZA loop makes the Yta7 substrate-binding pocket more open. Furthermore, the conserved Tyr in the ZA loop of human ATAD2 and other BRD-containing proteins is replaced by Ser in Yta7, and the conserved Tyr/Phe-Asn dipeptide motif in the BC loop is replaced by Ile–Gly in Yta7 (Fig. 2, *F* and *G*). These substitutions likely account for the observation that Yta7 binds to histones in a post-translational modification–independent manner (18). We next examined the binding affinity of Yta7 with the H3 tail (1–22 amino acids) either in the nonacetylated or in the acetylated form by AlphaScreen (amplified luminescent proximity homogeneous assay). We found that both peptides bound Yta7, but the binding signal of the nonacetylated peptide was five times stronger than that of the acetylated version (Fig. 2*H*). Structures of the Yta7 BRD bound to both forms of the peptide would be required to understand the different affinities.

Because BIM is near the BRD substrate-binding pocket and stabilizes the BRD–BIM spiral, we asked if BIM plays a role in histone binding. We generate three Yta7 constructs, one removing the BRD (ΔBRD, 954–1132 amino acids), one removing the BIM motif (ΔBIM, 320–350 amino acids), and one removing both BRD and BIM. We were able to purify Yta7_ΔBRD and Yta7_ΔBIM ΔBRD, but not Yta7_ΔBIM (Fig. S9*A*), suggesting that BIM (320–350 amino acids) is important for Yta7 folding. The AlphaScreen assay showed that neither Yta7_ΔBRD nor Yta7_ΔBIM ΔBRD bind the H3 peptide (Fig. S9*B*). We next made the maltose-binding protein (MBP)-fused individual domain constructs (6xHis-MBP-BIM, 6xHis-MBP-BRD, and MBP-BIM) and purified these proteins (Fig. S9*C*). AlphaScreen assay showed that BIM alone had weak binding signal to H3 peptide—the signal was above the MBP control but weaker than BRD alone (Fig. S9*D*). Furthermore, the binding signal of BIM and BRD together was stronger than BRD alone. These results suggest that BIM participates in the BRD recognition of the H3 tail. We note that the binding signal of BRD–BIM is an order of magnitude weaker than that of the Yta7 hexamer, probably because of the presence of multiple BRD–BIM domains in the full-length Yta7 hexamer that all contribute to H3 binding.

### The Yta7 AAA1 PLs spiral around the histone H3 tail

**Figure 3:**
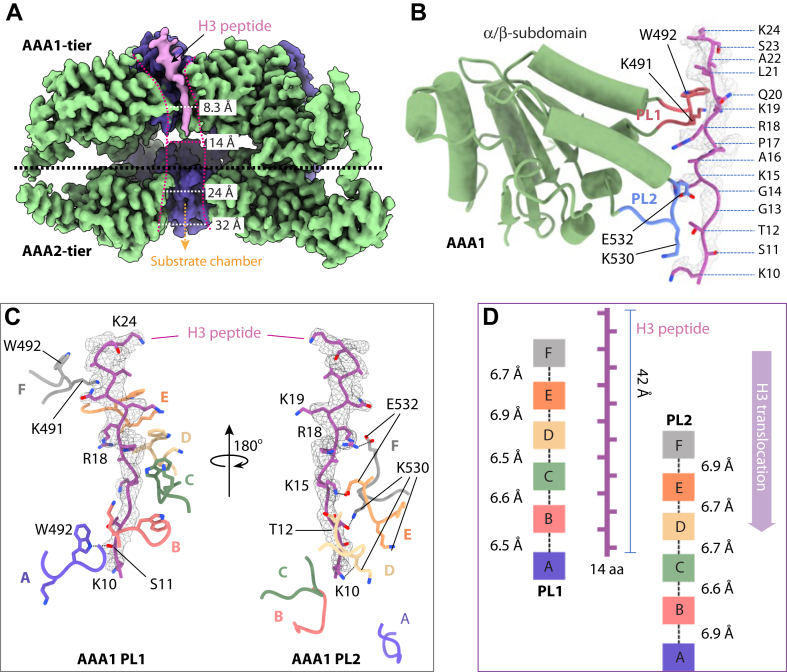
T**he H3 N-tail coordination in the central chamber of the ATPγS-bound Yta7 hexamer.***A*, a cut-open side view of the 3D map. The EM density of the trapped H3 N-tail is in *salmon*. *B*, structure of the AAA1 α/β subdomain with its PL1 (*yellow*) and PL2 (*cyan blue*) coordinating the modeled H3 tail. The H3 tail structure is in *sticks* superimposed with the EM density in *gray meshes*. *C*, close-up views of the interactions of H3 N-tail and the coordinating PL1 (*left*) and PL2 (*right*) of the AAA1 α/β subdomain. The EM density of H3 N-tail is superposed and is in *gray meshes*. Trp492 and Lys491 (show as *sticks*) from PL1 form a staircase around the H3 peptide. Hydrogen bonds are shown as *black dashed lines*, and the involved residues were labeled. The 7 Å axial distance is measured from PL1 Trp492 to PL2 Glu532. *D*, schematic of the H3-interacting double spiral formed by AAA1 PL1 and PL2, with interloop distances along the substrate axis shown based on the position of PL1 Trp492 (*left*) and PL2 Glu532 (*right*). N-tail, N-terminal peptide; PL, pore loop.

As described previously, in the ATPγS-bound state II where the BRD–BIM tier is largely disordered and forms a flat ring, the substrate entry is open, and there is a linear and continuous density with side-chain features in the Yta7 AAA1 chamber. The density best fits with the C-terminal 15 residues (Lys10–Lys24) of the added H3 tail (Figs. 1*C*, 3*A* and S10), but the best is not perfect for every side chain, indicating that a small population of the H3 peptides in the Yta7 may be out of register. The modeled H3 peptide is oriented with its N terminus pointing downward (Fig. 3*B*), suggesting that the N terminus of H3 first threads into the Yta7 central chamber, and that the unresolved N-terminal nine residues may have reached the AAA2 chamber and become disordered. Indeed, the AAA2 chamber is wider (24–32 Å) than the AAA1 chamber (8–14 Å) as it lacks the peptide-translocating PLs present in the AAA1 (Fig. 3*A*).

The modeled H3 peptide (Lys10–Lys24) is stabilized by PL1 and PL2 of the Yta7 AAA1 α/β subdomain (Fig. 3*B*). Specifically, six PL1 (one from each of the six subunits) surround the peptide like a right-handed staircase with six Trp492 contacting the peptide backbone (Fig. 3*C*). However, the lowest Trp492 of subunit A has weaker interaction with the H3 peptide because its side chain points away from the substrate, suggesting that the subunit A PL1 is about to disengage from the substrate. The six PL2 also form a right-handed spiral to surround the substrate peptide, but only four PL2 loops (of subunits C–F) contact the peptide, *via* hydrogen bonds between subunit F Glu532 and H3 Arg18, between subunit E Glu532 and H3 Arg15, and between subunit D Lys530 and H3 Lys10. The two PL2 of the lower subunits A and B are away from the H3 peptide. These substrate-interacting residues are conserved in the Abo1 structure, and mutations W345A and E385A in Abo1—equivalent to W492A and E532A in Yta7—diminished the histone manipulation function while not affecting the ATPase activity, underscoring the importance of these peptide-binding residues (15). The axial distance between the H3-interacting Trp492 in adjacent PL1s and between Glu532 in adjacent PL2s is around 6 to 7 Å, indicating a peptide-translocation step size of two amino acids (Fig. 3*D*). The aromatic PLs spiraling around the peptide substrate and the two-residue per translocation step are conserved in several well-characterized AAA+ protein unfoldases (12, 27, 28).

### The nucleotide-binding pattern suggests a sequential ATP hydrolysis mechanism

**Figure 4:**
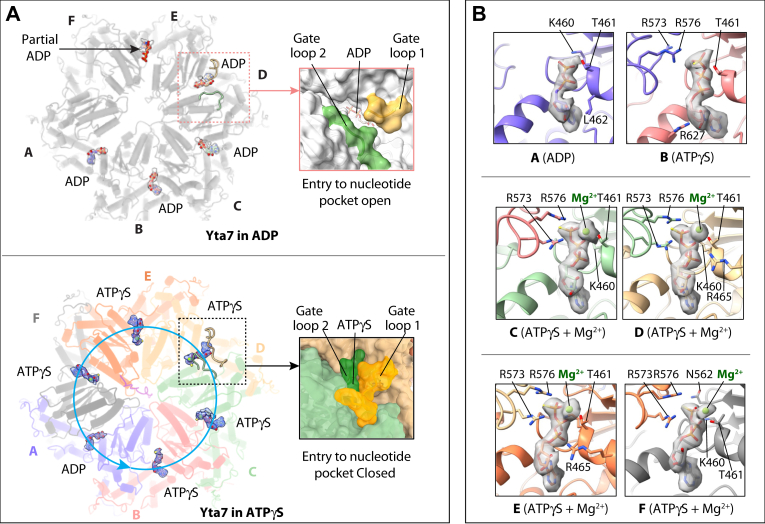
**Nucleotide binding in the ADP- and ATPγS-bound Yta7 structures.***A*, *top view* of the AAA1 tier of Yta7 in the ADP-bound state and ATPγS-bound state II. The bound nucleotides are in *sticks* superimposed with the EM map rendered in transparent surface views. The *arrowed cyan circle* indicates sequence ATP hydrolysis in the counterclockwise direction. The ADP/ATPγS-binding pocket in subunit D of Yta7 in the ADP- and ATPγS-bound states is shown in enlarged views on the *right*. The nucleotide entry/exit gate is open in the ADP-bound state but is closed by gate 1 and gate 2 loops in the ATPγS-bound state. *B*, detailed view of the nucleotide-binding site in each subunit in the ATPγS-bound Yta7 state II. ADP/ATPγS are shown in *sticks* with their respective EM densities superimposed in transparent *gray* surface view. The Mg^2+^ ion resolved in subunits C–F are in *green spheres*. The six EM densities are rendered at a same threshold. The residues that coordinate the nucleotide are in *sticks* and labeled.

The resolution of our cryo-EM maps was sufficient to identify the bound nucleotides (Fig. 4). We observed no nucleotide in the AAA2 tier in all Yta7 structures, consistent with the knowledge that the AAA2 is an inactive AAA+ fold. In the ADP-bound Yta7, we identified four ADP molecules in the AAA1 domains of subunits A–D, a partially occupied ADP in subunit E, and no ADP in subunit F (Fig. 4*A*). In the ATPγS-bound Yta7 state I, each AAA1 domain contains a nucleotide with density features consistent with ATPγS (Fig. S11*A*). However, in the ATPγS-bound Yta7 state II, five ATPγS molecules were identified in the AAA1 domains of subunits B–F. The EM density in the nucleotide-binding pocket of the lowest subunit A is consistent with an ADP, suggesting the ATPγS in this site has been hydrolyzed (Fig. 4). Interestingly, the entry to the nucleotide-binding pocket is largely open in the ADP-bound state but is closed in the ATPγS-bound state (Fig. 4*A*). The entry is controlled by two gating loops: gate loop 1 is formed by Pro403–Asn409, and gate loop 2 is formed by Met549–Arg552 that connects α3 and β4 of an adjacent AAA1. In the ATPγS-bound state, gate loop 1 of subunit D moves toward subunit C and gate loop 2 of subunit C clamps down to cover the entry path. These loops move away from their positions in the ATPγS-bound state to open the nucleotide entry path, perhaps facilitating nucleotide exchange in the ADP state.

In the ATPγS-bound state II structure, the five ATPγS molecules in subunit B–F are stabilized by Lys460 and Thr461 of the hosting subunit and Arg573 and Arg576 of the adjacent subunit (Fig. 4*B*). In subunit A, the ADP is stabilized only by Lys460 and Thr461 because Arg573 and Arg576 of the adjacent subunit are too far to interact. The EM density for the Mg^2+^ ion coordinating the ATPγS molecule is strong and well resolved in subunits C–F but weaker in subunit B (Fig. S11*B*). We expect that ATP hydrolysis occurs counterclockwise in the order of subunits A–B–C–D–E–F–A (Fig. 4*A*). In other words, the next subunit to hydrolyze ATP is B, and when subunit B takes on the pose of subunit A, its PL1 and PL2 will move down by ∼7 Å, thereby pulling the bound H3 peptide downward by two amino acids (Fig. 3*D*). The sequential ATP hydrolysis mode by the Yta7 hexamer is consistent with other well-characterized protein unfoldases (28, 31, 32, 33, 34, 35, 36, 37).

### Nucleosome binding flattens the Yta7 BRD spiral to a ring

**Figure 5:**
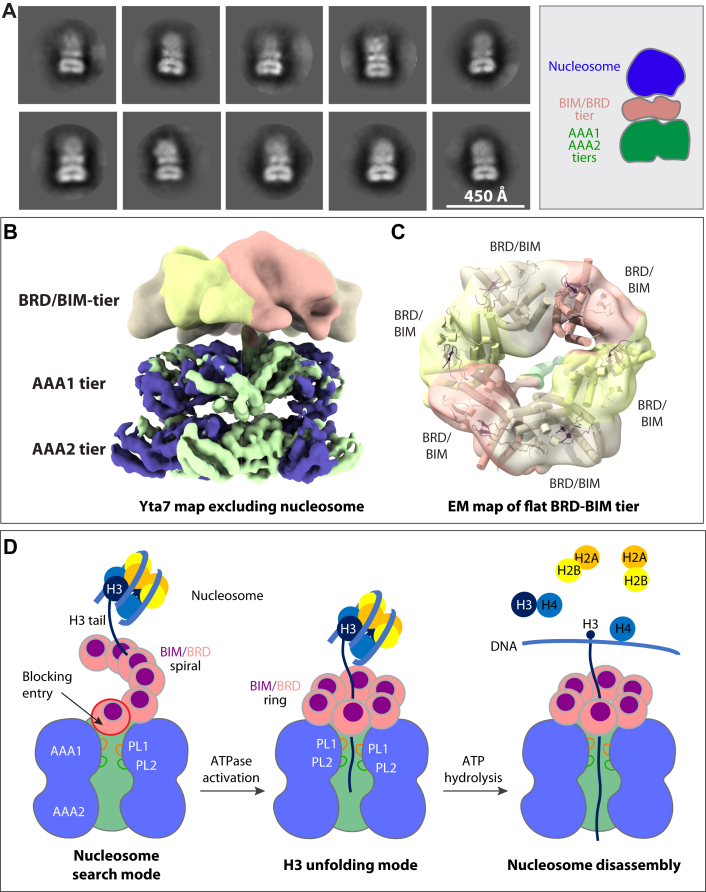
**Nucleosome binding and conformational changes of Yta7.***A*, selected 2D class averages of the Yta7–nucleosome complex, showing flexible association of the nucleosome. *B*, composite map of the Yta7 region by excluding flexible nucleosome in 3D reconstruction. The AAA+ core region (5.2 Å resolution) and the BRD–BIM tier (12 Å resolution) were separately refined and combined in Chimera. *C*, the EM density of the BRD–BIM tier superimposed with six copies of the BRD–BIM domains. This tier is in a flat-ring configuration. *D*, schematic model of the Yta7-mediated nucleosome removal from chromatin. BRD–BIM spiral represents an inactive model as the Yta7 central chamber is blocked by the proximal BRD–BIM. H3 tail recognition and nucleosome engages with Yta7, will convert the BRD–BIM spiral to a flat ring, and opens the entry to the Yta7 chamber. When the H3 tail is inserted into the AAA1 chamber, the PL1 and PL2 pull on H3 tail. Yta7 unfolds H3 in a hand-over-hand mechanism driven by ATP hydrolysis. Finally, H3 unfolding leads to nucleosome disassembly. BIM, BRD-interacting motif; BRD, bromodomain.

We next investigated how Yta7 interacts with the nucleosome. We coexpressed the yeast H2A, H2B, H3, and H4 in *Escherichia coli*, purified the histone octamer (Fig. S12*A*), and reconstituted the yeast nucleosome with 167 bp Widom 601 sequence by gradient dialysis (38). But Yta7 only weakly bound the reconstituted yeast nucleosome *in vitro*. We therefore employed the GraFix technique to stabilize the interaction with 0 to 0.15% glutaraldehyde (39) (Fig. S12*B*). 2D class averages of cryo-EM images of the cross-linked particles showed the nucleosome density on top of the Yta7 BRD tier (Figs. 5*A* and S12, *C* and *D*)). Further 3D classification did not result in EM maps with defined nucleosome density, indicating that the nucleosome binding on top of the Yta7 remains highly flexible in the cross-linked samples (Fig. S12*E*). We next excluded the nucleosome region and performed focused refinement to obtain a low-resolution 3D map of Yta7 (40). Further refinement focusing on the AAA core and the BRD tier separately yielded an improved composite map with the AAA region at 5.2 Å and the BRD tier at ∼12 Å resolution (Fig. 5, *B* and *C*). These analyses reveal that the Yta7 structure in the presence of nucleosome is highly similar to the Yta7 structure in the ATPγS and H3 peptide bound state I in which the BRD tier is a flat ring. Therefore, nucleosome appears to have stabilized the BRD–BIM tier in the flat-ring configuration, such that the peptide entry into the AAA1 central chamber is no longer blocked.

## Discussion

Yta7 is a recently identified chromatin segregase that disassembles nucleosomes in an S phase cyclin-dependent kinase–dependent manner to promote chromosome replication (16). We have solved the first nearly full-length structure of Yta7, revealing a similar hexameric architecture to the pombe homolog Abo1. The previous Abo1 structural study used an N-terminal truncated version, and the BRD was not modeled because of low resolution in that region (15). We discovered that the Yta7 BRD is not only expanded by two additional α-helices but also contains a much longer ZA loop and a distinct BC loop (Fig. 2). These structural features are likely responsible for our observation that the Yta7 BRD binds more tightly to unmodified histone H3 tail than to the acetylated version, although a structure of H3 N-tail-bound BRD is required to understand how the initial recognition occurs. We identified the novel BIM in the N-terminal region with a β-helix fold (Fig. 2, *B* and *C*). The BIM structurally mediates the BRD–BRD interaction on top of the AAA+ hexamer for nucleosome recognition, and we have demonstrated that BIM also contributes to the BRD binding and recognition of H3 N-tail peptide (Fig. S9).

In the ATPγS-bound state II structure, we found that the modeled H3 peptide (1–24 amino acids) has passed through the BRD tier and entered the AAA chamber, with the N-terminal region of the peptide (1–9 amino acids) apparently disordered inside the AAA2 chamber and the C-terminal region (10–24 amino acids) fully engaged by the AAA1 PLs 1 and 2 like a staircase (Fig. 3, *A* and *B*). The peptide orientation is consistent with the need for the N terminus of the H3 tail to thread first into the Yta7 for unfolding. The nucleotide–binding pattern and the peptide-translocation PL arrangement in the AAA1 tier support a counterclockwise sequential ATP hydrolysis cycle and a processive peptide unfolding mechanism of the Yta7 hexamer (Figs. 3, *C* and *D* and 4). Therefore, the peptide-translocation aspect of the Yta7 is similar to several other well-characterized protein unfoldases (28, 31, 32, 34, 35, 36).

The Yta7 AAA2 adopts an inactive α/β fold without a nucleotide-binding site and without substrate-binding PLs. The AAA2 domain is complete in our structure, and there is no loop density protruding to the central chamber to interact with the translocating substrate peptide. We suggest that the inactive AAA2 tier functions to stabilize the hexamer structure. However, the BRD is inserted in the AAA2. Therefore, AAA2 must also be important for organizing the unique three-tiered architecture of the Yta7.

Importantly, we found that the Yta7 BRD tier can exists in a spiral form or a flat-ring form (Figs. 1*C* and S6). In the spiral form, the substrate entry to the AAA chamber is blocked by the proximal BRD (Figs. 2, *A* and *D* and S6*A*), and upon nucleosome binding, the BRD tier is flattened into a closed ring, unblocking the peptide entry to the AAA chamber (Figs. 5, *B* and *C* and S6*A*). Based on these structural findings, we propose the following mechanism of nucleosome recognition and unfolding by the Yta7 hexamer (Fig. 5, D). We suggest that the Yta7 structure with a spiral BRD tier is in a nucleosome search mode because the substrate entry to the AAA chamber is blocked in this mode, and the extended BRD spiral may provide multiple BRD–BIM domains for a specific recognition of the H3 N-tail. The capture of an H3 N-tail will lead to nucleosome binding, which will in turn convert the BRD spiral into a flattened ring. The conversion opens the substrate entry to the AAA chamber and perhaps reduces the binding affinity of the BRD tier to the H3 peptide, leading to the release of the substrate from the BRD tier and threading of the peptide into the AAA chamber. Continued ATP hydrolysis in AAA motor then drives H3 peptide translocation, leading to the disassembly of the nucleosome.

In summary, our work has shed new lights on the Yta7-mediated nucleosome disassembly. Further studies are needed to understand how the disordered N-terminal region contributes the nucleosome binding, and how the BRD–BIM recognizes the H3 tail before delivering it to the AAA1 chamber for unfolding.

## Experimental procedures

### DNA construct generation

*S*. *cerevisiae YTA7* gene was amplified from yeast DNA genome and cloned into the integrated and galactose-inducible vector pRSII403 with an N-terminal 10xHis tag and 3xFLAG tag. The DNA sequences encoding the Yta7 BIM (320–350) and the BRD (956–1125) and fused with an N-terminal 6xHis-MBP tandem tag were cloned into a pETDuet1 vector (Novagen). The Yta7 BIM (320–350) was also cloned into a pET29a expression vector with an MBP fusion but without the 6xHis tag. Genes encoding *S. cerevisiae* histones H2A, H2B, H3, and H4 were cloned into and replaced the same *Xenopus laevis* histone regions in the polycistronic coexpression plasmid pET29a-YS14 (Addgene #66890). All plasmid constructs were verified by sequencing.

### Protein expression and purification

The pRSII403-Yta7 and pRSII403-Yta7 mutant plasmids were linearized and transformed into yeast strain ySK119 (W303 background) (41) to integrate in the HIS3 region. All the strains and plasmids used in this work are listed in Table S1. Yeast overexpression strains of WT and mutant Yta7 were grown in selective medium before being inoculated into 9 l of the YP-raffinose medium at 30 °C. Cells were grown to an absorbance of 1.0 at 600 nm and arrested for 3 h with 100 ng/ml of α-factor (GenScript). Then 2% (w/v) galactose was added to induce protein overexpression for 4 to 5 h at 30 °C. After induction, the cells were collected and washed with 200 ml ice-cold 25 mM Hepes–KOH (pH 7.6)/1 M sorbitol once, washed twice with lysis buffer (250 mM KCl, 25 mM Hepes–KOH [pH 7.6], 10% glycerol, 0.05% NP-40, 1 mM EDTA, and 4 mM MgCl_2_). The cell pellets were resuspended in the same cell volume of the lysis buffer containing two protease inhibitor tablets, and the suspensions were frozen drop by drop into liquid nitrogen. The frozen cells were crushed using a freezer mill (SPEX CertiPrep 6850 Freezer Mill) for 12 cycles, 2 min each at 15 cps with 2 min cooling in between. The crushed cellular powder was slowly thawed on ice overnight and then mixed into an equal volume of lysis buffer plus protease inhibitors. Insoluble material was removed by centrifugation in a Ti-45 rotor at 40,000× rpm for 1 h. The supernatant was incubated in batch mode with prewashed anti-FLAG M2 affinity gel (Sigma) for 4 h at 4 °C. After flow through, the beads were washed by 50 column volumes (CVs) of lysis buffer and then washed again by another 50 CV lysis buffer. Finally, proteins were eluted with 5 CV lysis buffer containing 0.3 mg/ml FLAG peptides. The WT and mutant Yta7 proteins were further purified by size-exclusion chromatography using a Superose 6 10/300 GL column (GE Healthcare) in a cold buffer (150 mM KCl, 25 mM Hepes–KOH [pH 7.6], 1 mM EDTA, 4 mM MgCl_2_, and 2 mM 2-mercaptoethanol). Protein concentration was determined using a Nanodrop device.

The MBP-fused Yta7-BIM and Yta7-BRD plasmids were transformed into *E. coli* BL21 (DE3), and the transformants were grown in 2 l of LB medium at 37 °C. When the cell density reached an absorbance of 0.8 at 600 nm, we induced the expression of the MBP-fused truncated proteins by adding 0.2 mM IPTG and continued the culture for 12 h at 16 °C. We then collected the cells, resuspended in buffer A (20 mM Hepes [pH 7.6], 150 mM NaCl, and 10% glycerol), and lysed the cells with a homogenizer (SPX Corporation). The lysate was centrifuged at 20,000*g* for 40 min, and the supernatant was collected and loaded into a 5 ml MBPTrap HP column (Cytiva). The MBP fusion protein was eluted using buffer A plus 10 mM maltose and was further purified by size-exclusion chromatography through a Superdex 200 column (GE Healthcare) in buffer A.

We transformed the modified pET29a-YS14 plasmids into the Rosetta 2 *E. coli* cells (Novagen) to make the yeast histone proteins. The transformant was inoculated into 6 l of LB medium and grown at 37 °C to an absorbance of 0.6 at 600 nm and then 0.3 mM IPTG was added to induce histone overexpression. After induction, cell culture was continued at 37 °C for 4 h. Then, the cells were harvested by centrifugation and resuspended in 240 ml (40 ml per 1 l *E. coli* culture) ice-cold high salt buffer (2 M NaCl, 25 mM Hepes [pH 7.5], 10% glycerol, and 1 mM 2-mercaptoethanol). Cells were lysed in the homogenizer, and the lysate was clarified by centrifugation at 40,000*g* for 1 h. We included 50 mM imidazole in the supernatant and loaded the solution into an FPLC with a 5 ml nickel– nitrilotriacetic acid affinity column. We washed the column with 20 CV of the high salt buffer and then eluted the protein by an imidazole gradient from 20 to 500 mM. The elution peak was collected, concentrated, and ran through a HiTrap Heparin HP column (Cytiva) in the high salt buffer to separate the soluble histone octamer. The corresponding fractions were concentrated to 2 mg/ml, and the sample quality was examined by SDS-PAGE gel.

### AlphaScreen assay

We performed AlphaScreen assays using a 6xHis detection kit (PerkinElmer). The biotinylated unmodified and the all-four-lysine acetylated H3 peptide (1–22 amino acids) were synthesized by GenScript. We individually mixed the purified 50 nM 10xHis-3xFlag-Yta7, 10xHis-3xFlag-Yta7ΔBRD, 10xHis-3xFlag-Yta7ΔBIMΔBRD, 6xHis-MBP-BIM, 6xHis-MBP-BRD, and the complex of 6xHis-MBP-BRD and MBP-BIM with 50 nM biotinylated and unmodified or acetylated H3 (1–22 amino acid) peptides, respectively, and incubated the mixtures on ice for 1.5 h with the streptavidin-coated donor beads (5 μg/ml) and nickel-chelated acceptor beads (5 μg/ml) in 50 mM Mops (pH 7.4), 100 mM NaCl, and bovine serum albumin (0.1 mg/ml). For nucleosome-binding assay, we used 50 nM 10xHis-3xFlag-Yta7 and 100 nM biotinylated human nucleosome. We incubated the mixture for 2 h on ice with the same concentration donor and acceptor beads. The donor beads contain a photosensitizer that converts ambient oxygen into short-lived singlet oxygen upon 680 nm light activation. When the acceptor beads are brought close enough to the donor beads by interaction between His-tagged proteins and biotinylated peptides, singlet oxygen can diffuse from the donor to the acceptor beads to transfer the energy to the thioxene derivatives of the acceptor beads, resulting in light emission at 520 to 620 nm range.

### ATPase assays

The Malachite Green Phosphate assay kit (Sigma–Aldrich) was used to measure the ATP hydrolysis rate of Yta7 present in different nucleotides. Yta7 was incubated in assay buffer (25 mM Hepes [pH 7.6], 2 mM MgCl_2_, 150 mM KCl, and 1 mM DTT) with or without histone-3 peptide (1–24 amino acids). Either 2 mM ATP or 2 mM ATPγS or 2 mM ADP (negative control) was added to initiate the reaction at room temperature for 1 h. Absorbance at 620 nm was measured in a Molecular Devices SpectraMax M2e microplate reader.

### Yeast nucleosome core particle reconstitution

We used the 167 bp Widom 601 DNA sequence to reconstitute the yeast nucleosomes. The W601 DNA was amplified by PCR and purified on a HiTrap Q column. After ethanol precipitation, the DNA was resuspended in 25 mM Hepes (pH 7.5), 2 M NaCl, and 1 mM DTT. The nuclear pore complexes were reconstituted following the established salt gradient dialysis method (38). Briefly, the W601 DNA and purified histone octamer were mixed in a 1.1:1.0 M ratio, each 100 μl mixture was put in a 0.5 ml Slide-A-Lyzer MINI Dialysis unit (10 kDa cutoff; Thermo Scientific), and first dialyzed for 7 to 8 h into 500 ml buffer A (20 mM Tris–HCl [pH 8.0], 1 M NaCl, 1 mM EDTA, and 1 mM DTT) at 4 °C. The units were then dialyzed into 500 ml buffer B (20 mM Tris–HCl [pH 8.0], 0.6 M NaCl, 1 mM EDTA, and 1 mM DTT) overnight. Finally, the dialysis units were moved into a fresh low salt buffer C (20 mM Tris–HCl [pH 8.0], 50 mM NaCl, 1 mM EDTA, and 1 mM DTT) and dialyzed for additional 2 to 3 h. The reconstitution results were analyzed on 6% native PAGE, and the nucleosome sample was concentrated to 1 mg/ml for future use.

### Sample preparation for cryo-EM

Purified Yta7 at ∼10 mg/ml was incubated with either 2 mM ADP or with 2 mM ATPγS plus unmodified H3 peptides (1–24 amino acids) (GenScript) in twofold molar excess to Yta7 hexamer. The mixtures were subsequently incubated at 4 °C for 2 h. We added 0.025% octyl β-d-glucoside into the mixture right before cryo-EM grid preparation, which alleviated the preferred particle orientation issue. We applied 3 μl sample droplets two times onto Quantifoil grids (copper R 2/1, 300 mesh) that were freshly glow discharged in an Ar/O_2_ mixture for 30 s using a Gatan 950 Solarus plasma cleaning system with the power set to 15 W. EM grids were blotted by qualitative cellulose filter paper (Ted Pella, Inc) for 3 s after each sample application with the blotting force set to 3. The blotted EM grids were plunge-frozen in liquid ethane cooled by liquid nitrogen in an FEI Vitrobot Mark IV with the sample chamber set to 8 °C and 100% humidity.

To improve Yta7 binding with nucleosome, we used the established gradient fixation (GraFix) method to prepare the Yta7–nucleosome complex (39). We incubated 2 μM Yta7 with 3 μM nucleosome and 2 mM ATPγS for 1 h on ice in a GraFix buffer (20 mM Hepes, pH 8.0, 150 mM NaCl, 1 mM MgCl_2_, and 1 mM DTT) and then loaded the sample onto a 12 ml linear 10 to 30% (v/v) glycerol gradient tube in the GraFix buffer supplemented with 0 to 0.15% EM-grade glutaraldehyde. The gradient tube was centrifuged at 35,000 rpm in an SW41 rotor (Beckman Coulter) at 4 °C for 16 h. The solution was fractionated from the bottom to top using an ÄKTA start chromatography system (GE Healthcare Life Sciences). Peak fractions were analyzed by SDS-PAGE, and the fractions containing the desired complexes were pooled and buffer exchanged to remove the glycerol and concentrated to approximately 0.5 mg/ml. For cryo-EM, we applied 3 μl of cross-linked Yta7–nucleosome complex onto Quantifoil EM grids (gold R 2/1, 300 mesh) that were plasma cleaned for 30 s in a Gatan Solarus. The EM grids were blotted for 3 s with a piece of filter paper. The grids were vitrified by plunging them into liquid ethane in an FEI Vitrobot Mark IV that was set to 8 °C and 100% humidity. The cryo-EM grids were stored in liquid nitrogen until data collection.

### Cryo-EM data collection

Cryo-EM datasets were collected in a Titan Krios electron microscope (Thermo Fisher Scientific) operated at a high tension of 300 kV with a K3 summit direct electron detector (Gatan). All EM images were recorded automatically using the SerialEM program (42) in a multihole mode by image shift with a maximum shift of 2.5 μm, at a nominal magnification of 105,000×, and with the objective lens defocus value set to vary from –1.0 to –2.0 μm. The electron detector was operated in the super-resolution counting mode, acquiring 75 individual frames during a 1.5 s exposure. With a dose rate of 0.88 electrons per Å^2^ per second per frame, the cumulative exposure dose was 65 e^−^/Å^2^. The calibrated physical pixel size was 0.828 Ǻ for all digital micrographs.

### Image processing

The dataset of ADP-bound Yta7 contained 12,456 movie stacks. The full dataset was split into five subgroups and imported to Relion-3.1 (https://relion.readthedocs.io/en/release-3.1/) (43). Micrographs in each subgroup were drift-corrected with electron-dose weighting using MotionCor2 (version 1.3.2) (https://github.com/singleparticle/MotionCor2) (44). The defocus value of each micrograph was estimated by CTFFIND4 (https://grigoriefflab.umassmed.edu/ctffind4) (45). We first manually picked a small set of particle images to generate templates that were subsequently used for autopicking (160–250 Ǻ diameter) in Relion-3.1 (43). A total of 3,130,724 particles were picked using a 400-pixel box size, which was scaled down by a factor of 4 using Relion-3.1. These particles were imported into cryoSPARC (version 3.2.0) (https://cryosparc.com/) to perform three rounds of 2D classification and to calculate a starting 3D model (46). The selected good 2D classes contained 593,691 particle images. These particle images were converted to the RELION format using UCSF PyEM (version 0.5) (https://github.com/asarnow/pyem). We classified the particle set into three 3D classes. Two major 3D classes with 299,123 and 215,755 particles, respectively, were combined to refine a 3D map at 3.3 Å resolution. After per-particle contrast transfer function refinement and Bayesian polishing, the final 3D map reached an overall resolution of 3.1 Å, based on the gold-standard Fourier shell correlation curve of two half maps at the standard threshold of 0.143 (47). The local resolution of the BRD–BIM tier was low because of the apparent mobility of this region. We next used cryoDRGN (https://cryodrgn.cs.princeton.edu/) (48) to discern the conformational heterogeneity of the BRD–BIM tier and derived five 3D maps with observable BRD–BIM domains ranging from zero to four. The EM map with three BRD–BIM domains at 3.9 Å resolution was combined with the AAA region of the aforementioned 3.1 Å resolution EM map to generate a composite 3D map (Fig. S2).

The dataset of the ATPγS- and H3 tail–bound Yta7 contained 12,085 movie stacks. These images were processed similarly as described previously. A total of 4,062,171 particles were picked. The selected good 2D classes contained 572,451 particle images. After 3D classification in Relion-3.1, the major 3D class with 431,065 particle images was used for further refinement, leading to a 3D map with bound H3 peptide at an estimated resolution of 3.3 Å. Further per-particle contrast transfer function refinement and Bayesian polishing improved the map to 3.0 Å. We also employed cryoDRGN to analyze the conformations of the BRD–BIM tier in the dataset. One 3D class showed the BRD–BIM tier in a flat-ring configuration. Focused refinement on the BRD–BIM tier led to a low-resolution 3D map at 9.7 Å (Fig. S5). A second 3D class with 109,554 particle images was also selected for further refinement, leading to a 3D map at 5.6 Å resolution. This map had a spiral BRD–BIM tier. Local resolution 3D maps were calculated using ResMap (https://resmap.sourceforge.net/) (49). We assigned the 3D map with the spiral BRD–BIM tier as Yta7 ATPγS state I, the composite 3D map with the flat BRD–BIM tier as Yta7 ATPγS state II, and the 3.0 Å resolution map of AAA+ region as the core of the Yta7 ATPγS state II (Fig. S5).

The dataset of the cross-linked Yta7–nucleosome complex contained 14,937 movie stacks and was processed mostly in the cryoSPARC program. The autopicked particle dataset contained 3,152,817 particle images. Three rounds of 2D classification yielded class averages in which the Yta7 region had well-defined density features, but the top nucleosome region was blurry and featureless. We selected a dataset of 895,586 particle images belonging to 2D classes that had stronger nucleosome density. After *ab initio* 3D reconstruction and 3D classification, we obtained a low-resolution 3D map. But the nucleosome region of the map had little structural feature (Fig. S12*E*), indicating that the nucleosome is mobile when bound to Yta7. In an effort to improve the nucleosome density, we downscaled the particle images by a factor of 4 (to a pixel size of 3.312 Å) and reconstructed a 3D map with 213,712 particle images. However, the top nucleosome density was still featureless. Further heterogenous refinement failed to improve the nucleosome density. We next performed three-dimensional variability analysis as implemented in cryoSPARC. The results showed no dominant 3D classes, indicating that the nucleosome is continuously flexible on Yta7. Finally, we focused on Yta7 by masking out the nucleosome region and obtained a 14 Å EM map for the Yta7 hexamer region. We next performed focused refinement on the AAA core region and the BRD–BIM tier separately. The Yta7–nucleosome complex particles were re-extracted using a box size of 320 pixels (0.828 Å/pix), and the nucleosome region was subtracted from the particle images. We obtained an EM map of the AAA+ core at 5.2 Å and an EM map of the BRD–BIM tier at 12 Å resolution. These two maps were combined in ChimeraX to generate a composite Yta7 3D map (Fig. S12*E*).

### Atomic model building and refinement

We first used the Robetta server (50) to generate 200 homology models for Yta7 based on the pombe Abo1 structure (Protein Data Bank ID: 6JQ0) and selected the top five scoring models for model fitting and refinement. The starting Yta7 model was split into the AAA1, AAA2, and the C-terminal helical regions, and rigid body docked individually into the ADP-bound Yta7 3D map using UCSF Chimera (51). We then manually adjusted and built flexible linker regions and ADP molecules in our high-resolution EM map in Coot (https://www2.mrc-lmb.cam.ac.uk/personal/pemsley/coot/) (52). The first BRD domain and corresponding BIM motif was built based on the high-resolution density region, and the other two BRD–BIM domains were rigid body docked into the lower resolution density in Chimera. The resulting model was subjected to several iterations of real-space refinement with rigid body fitting and secondary structure restraints using the PHENIX package (http://www.phenix-online.org/) (53) and manual adjustment in Coot (52).

The Yta7 map in the ATPγS state I was similar to the ADP-bound Yta7 EM map, but the resolution was too low for atomic modeling. Therefore, we only deposited the 3D map to the Protein Data Bank and used the rigid-body docked atomic model for figure presentation. For the higher resolution 3D map of the AAA+ core region of Yta7 in the ATPγS state II, individual ATPase domains extracted from the ADP-bound Yta7 atomic model were docked into the EM map using USCF chimera (51). We manually adjusted the linker regions of the atomic model in Coot (52). Because the top BRD–BIM tier was at a lower resolution, we used AlphaFold-Multimer (54) to predict an atomic model of the hexameric BRD–BIM tier and docked the predicted model in the EM map. The H3 N-terminal peptide (10–24 amino acids), ADP, ATPγS, and magnesium ions were manually built into the EM map. The full atomic model was refined in real space in PHENIX (53). The final atomic model fits the 3D map density very well. We performed rigid-body docking in the composite Yta7 EM map derived from the cross-linked Yta7–nucloesome dataset, by using the atomic model of Yta7 in the ATPγS state II. Both the original maps and the maps sharpened by DeepEMhancer (https://github.com/rsanchezgarc/deepEMhancer) (55) were used for atomic model refinement. The UCSF ChimeraX was used to visualize the structure and prepare the figures (56). The statistics of model refinement are shown in Table S2.

## Data availability

The cryo-EM 3D maps of the *S. cerevisiae* Yta7 bound to ADP and Yta7 bound to H3 tail and ATPγS have been deposited in the Electron Microscopy Data Bank with accession codes EMD-26695, EMD-26696, EMD-26697, and EMD-26682. The corresponding atomic models have been deposited in the Protein Data Bank with accession codes 7UQI, 7UQJ, and 7UQK.

## Supporting information

This article contains supporting information.

## Conflict of interest

The authors declare that they have no conflicts of interest with the contents of this article.

## Supporting information

Supporting infomation
