# Supplementary material for: The Saccharomyces cerevisiae Yta7 ATPase hexamer contains a unique bromodomain tier that functions in nucleosome disassembly
Source: J Biol Chem. 2022 Dec 30;299(2):102852. doi: 10.1016/j.jbc.2022.102852 (PMC9898759; doi:10.1016/j.jbc.2022.102852)
Supplement: Supporting infomation [file mmc1.docx]

*Supporting information for*

The *S. cerevisiae* Yta7 ATPase hexamer contains a unique bromodomain tier that functions in nucleosome disassembly

By Feng Wang, Xiang Feng, Qing He, Hua Li, Huilin Li

This file contains 2 supplemental tables and 12 supplemental figures.

**Table S1. List of strains and plasmids used in this study**

| **Stains and plasmids** | **Description** | **Reference** |
| --- | --- | --- |
|  | **Yeast strains** |  |
| ySK119 | MATa pep4::unmarked bar1::hisG UBA3::pGAL1/10-�1-95- CLB5-FLAG+CDC28-His | (​1​) |
| yHL_Yta7 | MATa pep4::unmarked bar1::hisG UBA3::pGAL1/10-�1-95- CLB5-FLAG+CDC28-His:: pGAL1/10-His10-Flag3-Yta7 | This study |
| yHL_Yta7�BRD | MATa pep4::unmarked bar1::hisG UBA3::pGAL1/10-�1-95- CLB5-FLAG+CDC28-His:: pGAL1/10-His10-Flag3-Yta7(954-  1132 aa deletion) | This study |
| yHL_Yta7�BIM� BRD | MATa pep4::unmarked bar1::hisG UBA3::pGAL1/10-�1-95- CLB5-FLAG+CDC28-His:: pGAL1/10-His10-Flag3-Yta7(320-350  aa/954-1132 aa deletion) | This study |
|  | ***E. coli* strains** |  |
| BL21 (DE3) | F-, *omp*T hsdSB(rB-mB-)gal, dcm(DE3) | Life Technologies |
| Rosetta2(DE3) | F-, *omp*T hsdSB(rB-mB-)gal, dcm(DE3) pRARE2(Cam^R^) | Life Technologies |
|  | **Plasmids** |  |
| pRSII403 | Yeast integrating vector with HIS3 selectable marker, Ap^R^ | Addgene |
| pRSII403-Yta7 | pRSII403 harboring full-length Yta7 with an N-terminal His tag and 3xFlag tag, to produce a Yta7 overexpression yeast strain, Ap^R^ | This study |
| pRSII403-  Yta7�BRD | pRSII403 harboring Yta7 (954-1132 aa deletion) with a N-  terminal His tag and 3x Flag tag, to produce a Yta7 overexpression yeast strain, Ap^R^ | This study |
| pRSII403-  Yta7�BIM  �BRD | pRSII403 harboring Yta7 (320-350 aa deletion and 954-1132 aa deletion) with a N-terminal His tag and 3x Flag tag, to produce a Yta7 overexpression yeast strain, Ap^R^ | This study |
| pETDuet1 | Coexpression of multiple target genes in *E. coli*, Ap^R^ | Nonagon |
| pETDuet1-His- MBP-BRD (956-  1125) | pETDuet1 harboring a His-MBP-BRD fragment to over-express recombinant protein in *E. coli*, Ap^R^ | This study |
| pETDuet1-His- MBP-BIM (320-  350) | pETDuet1 harboring a His-MBP-BIM fragment to over-express recombinant protein in *E. coli*, Ap^R^ | This study |
| pET29a | Overexpression of the target gene in *E. coli*, Kan^R^ | Addgene |
| pET29a-MBP- BIM (320-350) | pET29a harboring an MBP-BIM fragment to over-express recombinant protein in *E. coli*, Kan^R^ | This study |
| pET29a-YS14 | Polycistronic coexpression vector to express *Xenopus laevis*  histones in *E. coli*, Kan^R^ | Addgene |
| pET29a-Yeast- Histones | Polycistronic coexpression vector harboring *Saccharomyces cerevisiae* histones in *E. coli*, Kan^R^ | This study |

**Table S2. Cryo-EM data collection, refinement, and validation statistics**

|  | #1 Yta7_ADP (EMDB-26697) (PDB ID 7UQK) | #2 Yta7_ADP composite map (EMDB-26695) (PDB ID 7UQI) | #3 Yta7_ ATPγS state II core region (EMDB-26696) (PDB ID 7UQJ) | #4 Yta7_ ATPγS state I  (EMDB-26682) |
| --- | --- | --- | --- | --- |
| **Data collection and processing** | | | | |
| Microscope | TFS Titan Krios | | TFS Titan Krios | |
| Magnification | 105,000 | | 105,000 | |
| Voltage (kV) | 300 | | 300 | |
| Detector | K3 | | K3 | |
| Total Electron exposure (e-/Å2) | 65 | | 65 | |
| Exposure rate (e-  /pixel/sec) | 30 | | 30 | |
| Energy filter slit width (eV) | 20 | | 20 | |
| Number of frames | 75 | | 75 | |
| Defocus range (μm) | –1.0 to –2.0 | | –1.0 to –2.0 | |
| Data acquisition software | SerialEM | | SerialEM | |
| Pixel size (Å/pixel) | 0.828 | | 0.828 | |
| Symmetry imposed | C1 | | C1 | |
| Micrographs collected | 12456 | | 12085 | |
| Initial particle images (no.) | 3,130,724 | | 4,062,171 | |
| Final particle images (no.) | 514,878 | 514,878 | 431,065 | 109,554 |
| Map resolution (Å) |  |  |  |  |
| FSC 0.5 | 4.1 Å (unmasked)  3.5 Å (masked) | 6.7 Å (unmasked)  4.3 Å (masked) | 3.8 Å (unmasked)  3.3 Å (masked) | 9.7 Å (unmasked)  7.5 Å (masked) |
| FSC 0.143 | 3.6 Å (unmasked)  3.1 Å (masked) | 4.3 Å (unmasked)  3.9 Å (masked) | 3.3 Å (unmasked)  3.0 Å (masked) | 7.2 Å (unmasked)  4.5 Å (masked) |
| Resolution range (local) | 2.7-5.4 Å | 2.7-9.3 Å | 2.5-4.3 Å | 4.1-12.9 Å |
| 3DFSC Sphericity value | 0.972 | 0.972 | 0.969 | 0.905 |
| **Refinement** | | | | |
| Initial model used (PDB code) | 6JQ0 | 6JQ0 | 6JQ0 |  |
| Refinement package | Phenix (Adams et al. 2010) | | |  |
| Model resolution (Å) | 3.2 | 3.2 | 2.9 |  |
| FSC threshold | 0.5 | 0.5 | 0.5 |  |
| Map sharpening *B* factor (Å2) | -102.3 | -109.7 | -94.4 |  |
| Model composition |  |  |  |  |
| Non-hydrogen atoms | 29141 | 32427 | 27985 |  |
| Protein residues | 3631 | 4025 | 3495 |  |
| Ligands | 5 | 5 | 10 |  |
| *B* factors (Å2) |  |  |  |  |
| Protein mean (min/max) | 73.84 (29.70/171.32) | 99.41 (57.58/332.33) | 34.53 (7.01/86.55) |  |
| Ligand mean (min/max) | 69.92 (38.98/151.87) | 87.91 (46.10/173.37) | 30.65 (20.35/41.62) |  |
| R.m.s. deviations |  |  |  |  |
| Bond lengths (Å) | 0.006 | 0.008 | 0.010 |  |
| Bond angles (°) | 1.240 | 1.315 | 1.057 |  |
| Validation |  |  |  |  |
| MolProbity score | 1.76 | 1.81 | 1.77 |  |
| Clashscore | 5.28 | 6.83 | 7.46 |  |
| Poor rotamers (%) | 0.12 | 0.41 | 0.61 |  |
| CCvolume/CCmask | 0.74/0.77 | 0.74/0.72 | 0.79/0.83 |  |
| CaBLAM Outliers (2) | 3.63 | 3.46 | 3.07 |  |
| EMRinger Score (3) | 2.08 | 1.71 | 2.57 |  |
| Ramachandran plot |  |  |  |  |
| Favored (%) | 92.35 | 93.36 | 94.76 |  |
| Allowed (%) | 7.65 | 6.64 | 5.18 |  |
| Disallowed (%) | 0.00 | 0.00 | 0.06 |  |


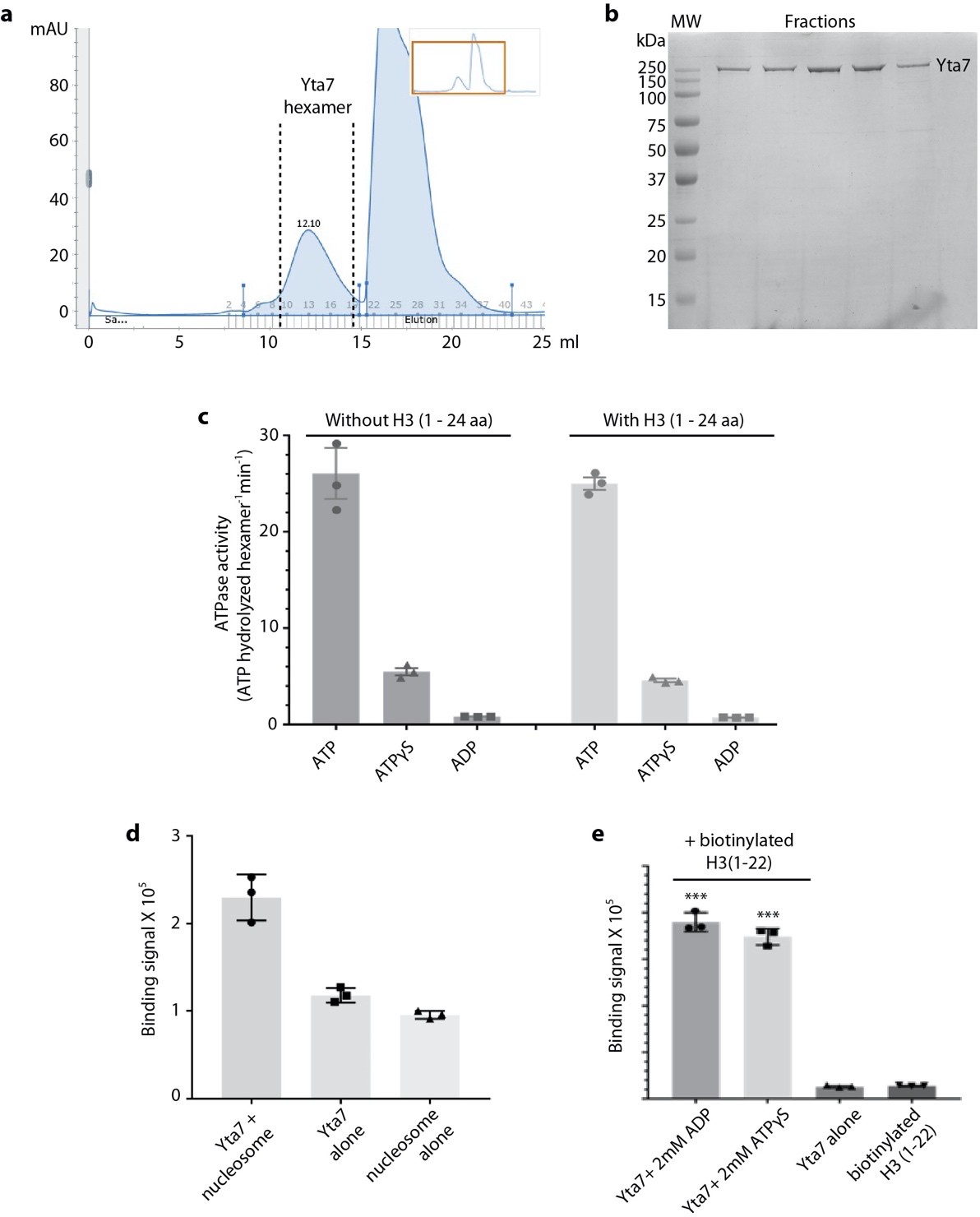


**Figure S1. Purification and characterization of the Yta7 hexamer. a,** Size exclusion chromatographic profile of purified Yta7 over a Superose 6 10/300 GL column. The Yta7 hexamer peak is indicated by two dash vertical lines. **b,** SDS-PAGE gel of the corresponded fractions from a. **c,** ATP hydrolysis activity by Yta7 in the absence or presence of the H3 (1 – 24 aa) peptide. The assay measured the released phosphate from nucleotide hydrolysis by Yta7. Error bars represent the standard error of three independent experiments. **d,** Binding of Yta7 to biotin-labeled human nucleosome as measured by AlphaScreen assay. **e**, AlphaScreen assay for binding of biotinylated H3 (1-22 aa) to Yta7 in the present of ADP or ATPγS. The binding is unaffected using ADP or ATPγS. All values represent means ± SD obtained from three independent experiments performed in triplicate. *** significantly different from Yta7 or H3 (1- 22) control (P ≤ 0.001, one-tailed t-test), ** (P ≤ 0.01, one-tailed t-test).


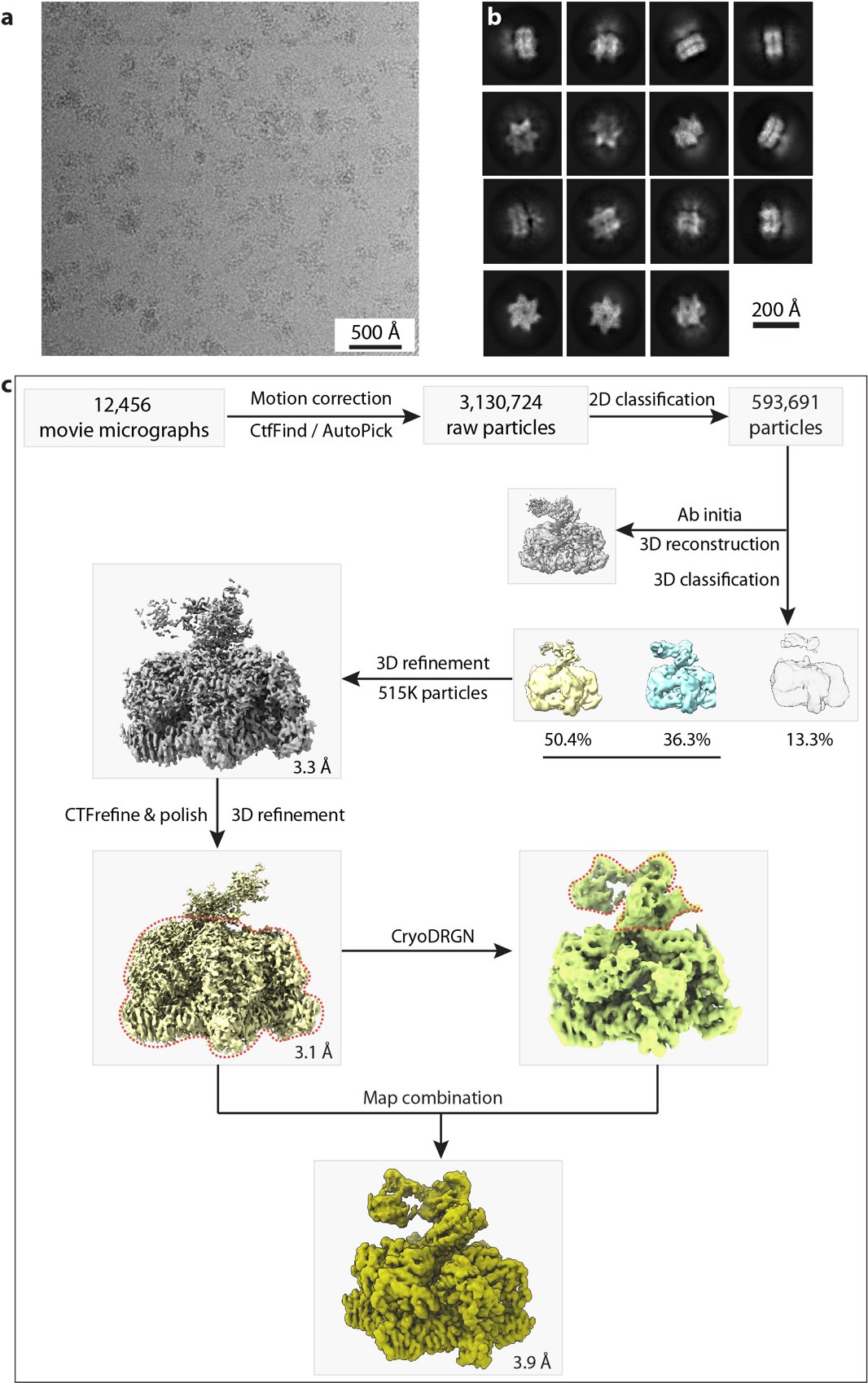


**Figure S2. Workflow of cryo-EM data processing and 3D reconstruction of Yta7 in the ADP state. a,** A typical raw micrograph. A total of 12,456 such micrographs were recorded in this study. **b,** Selected 2D class averages. **c,** Workflow of cryo-EM data processing of purified Yta7 in the presence of ADP.


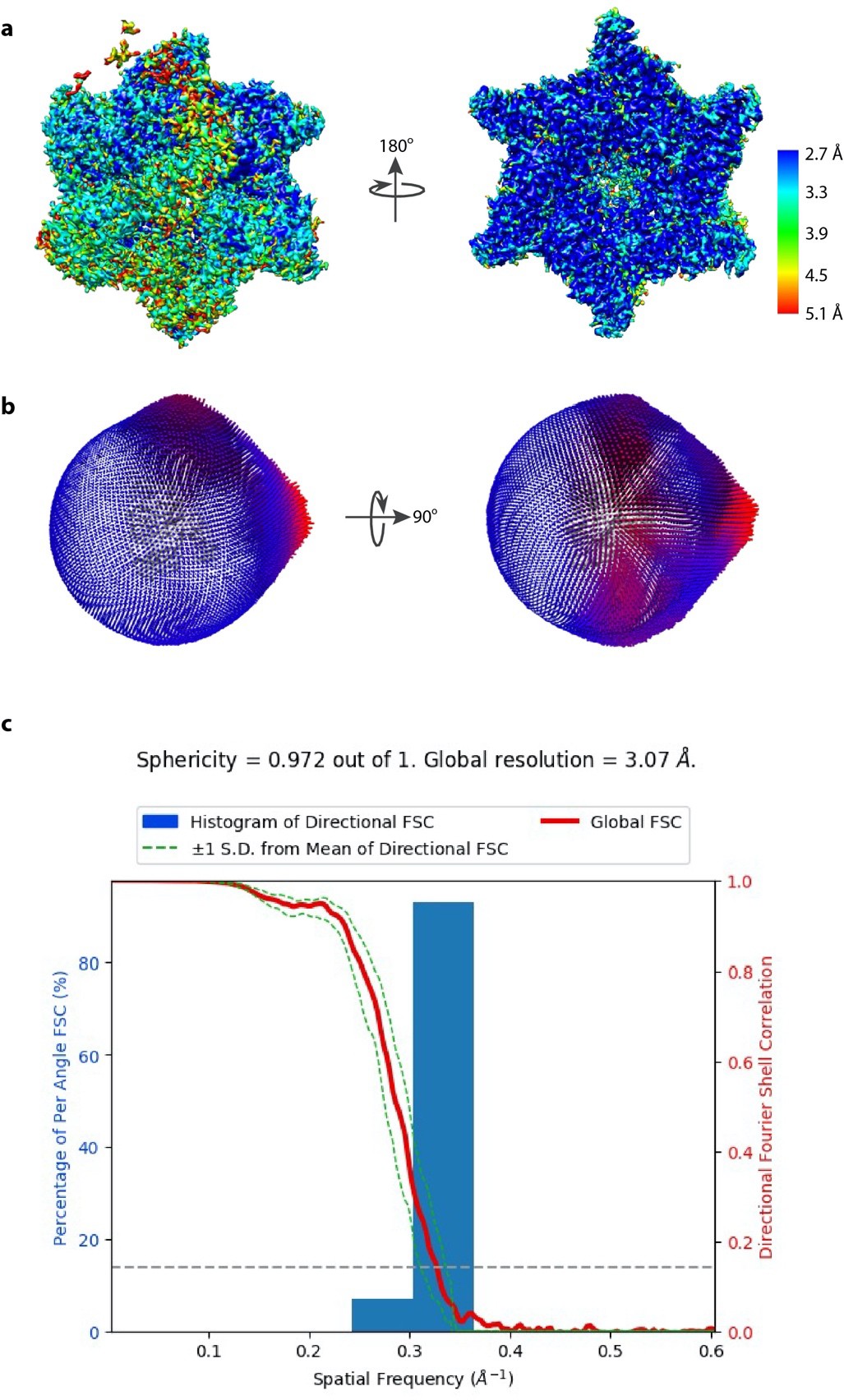


**Figure S3. Resolution estimation of the 3D map of the ADP-bound Yta7 hexamer. a**, Color coded local resolution map the ADP-bound Yta7 EM map. **b**, Angular distribution plot of particles used in the final 3D reconstruction. **c**, The directional anisotropy of the ADP-bound Yta7 EM map as quantified by the 3D-FSC server (https://3dfsc.salk.edu/). The EM map has a good anisotropic property with a sphericity of 0.972.


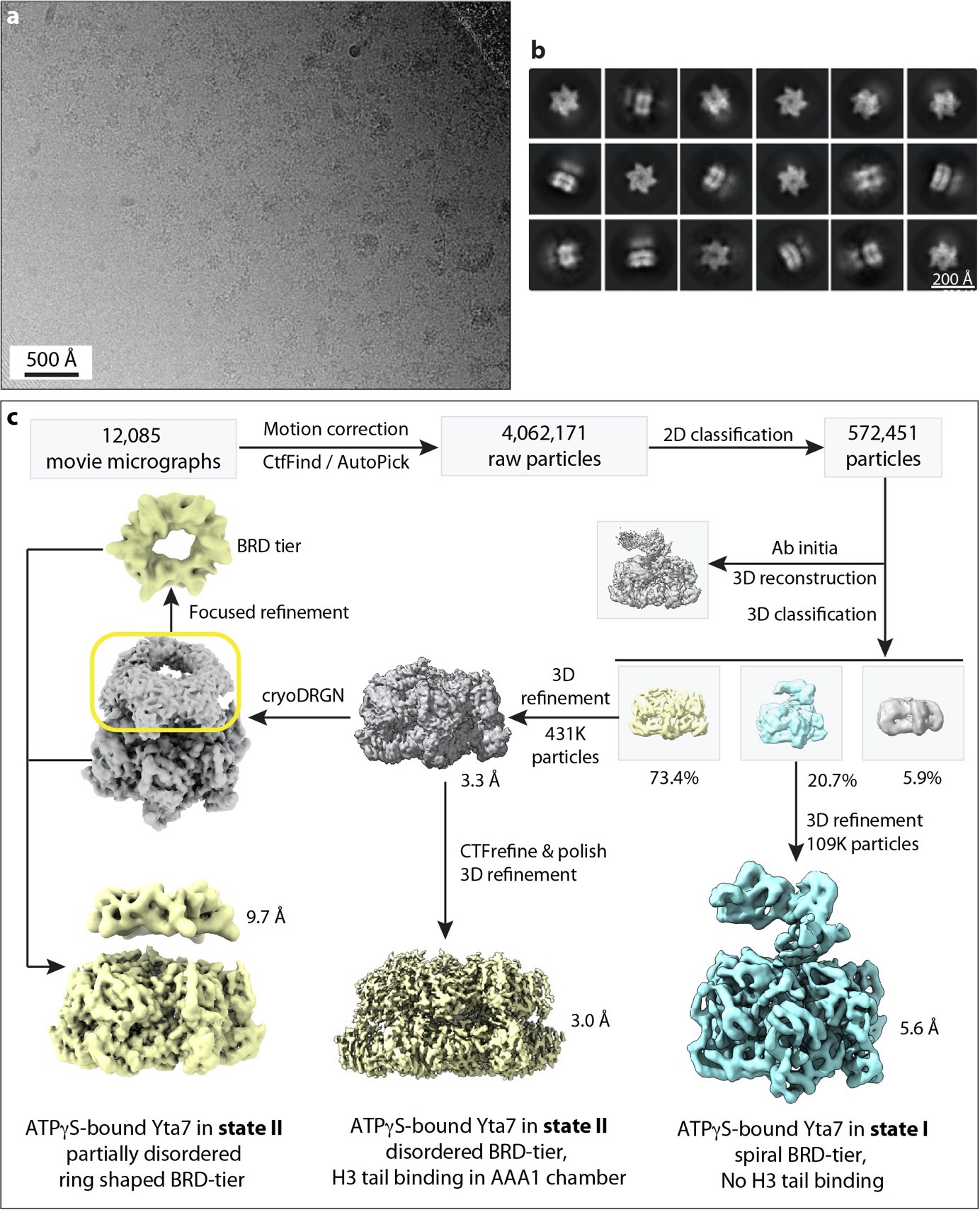


**Figure S4. Workflow of cryo-EM data processing and 3D reconstruction of Yta7 bound to ATPγS and an H3 peptide. a,** A typical raw micrograph. A total of 12,085 such micrographs were recorded in this study. **b,** Selected 2D class averages. **c,** Workflow of cryo-EM data processing using a combination of Relion, cryoSPARC, and cryoDRGN.


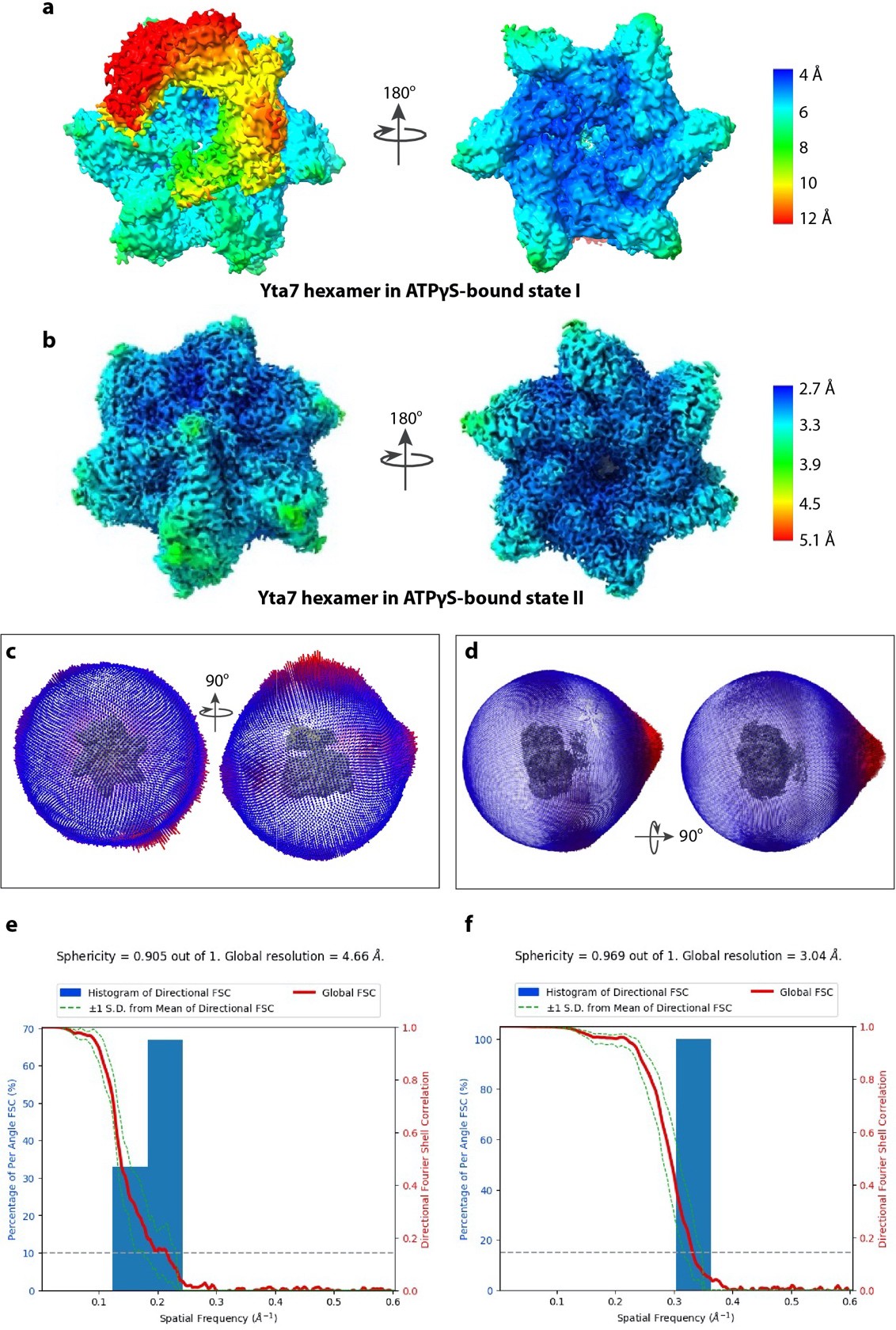


**Figure S5. Resolution estimation of the EM maps of ATPγS -bound Yta7 in states I and II. a-b**, Colored coded local resolution map of the EM maps of the ATPγS-bound Yta7 in state I (a) and state II (b, bound to H3 tail). **c-d**, Angular distribution plot of particles used for the final 3D refinement and reconstruction of Yta7 in ATPγS state I (c) and state II (d). **e-f,** The directional anisotropy of the EM map of ATPγS-bound Yta7 in state I (e) and state II (f) as quantified by the 3D-FSC server (https://3dfsc.salk.edu/). The 3D maps have good anisotropic property with a sphericity of 0.905 and 0.969, respectively.


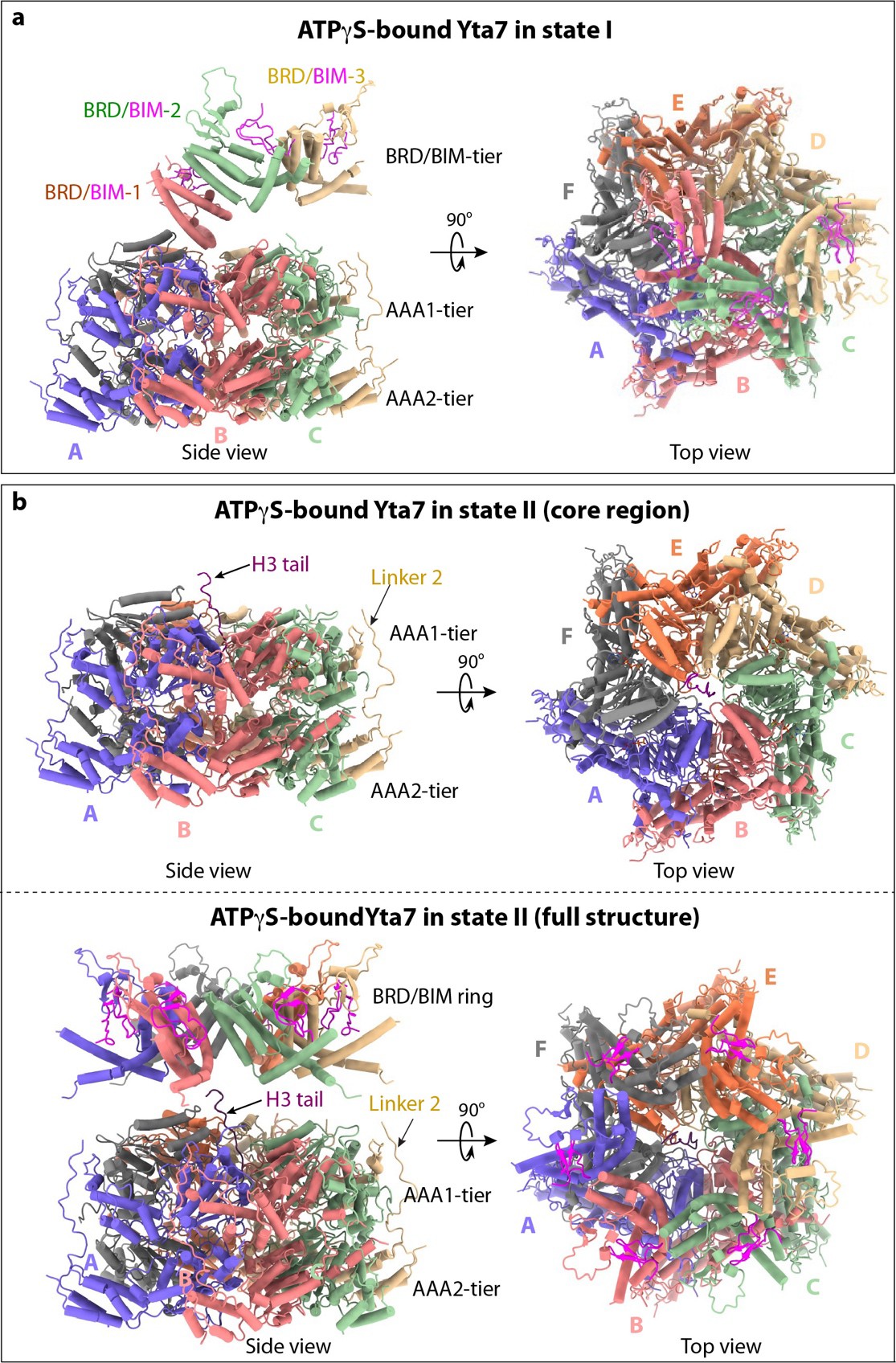


**Figure S6. Comparison of ATPγS-bound Yta7 in states I and II. a,** Cartoon views of the structure of ATPγS-bound Yta7 in state I in which the top BRD/BIM-tier is partially ordered, with the three BRD/BIM resolved and modeled. No H3 peptide was observed in this state, because the proximal BRD/BIM-1 blocks the peptide entry to the central chamber. **b,** Cartoon views of the core region (upper) and the full structure (lower) of ATPγS-bound Yta7 in state II. The BRD/BIM-tier resolved in a lower resolution EM map is a flat ring. Subunits are individually colored. H3 peptide is resolved in the AAA1 chamber in state II.


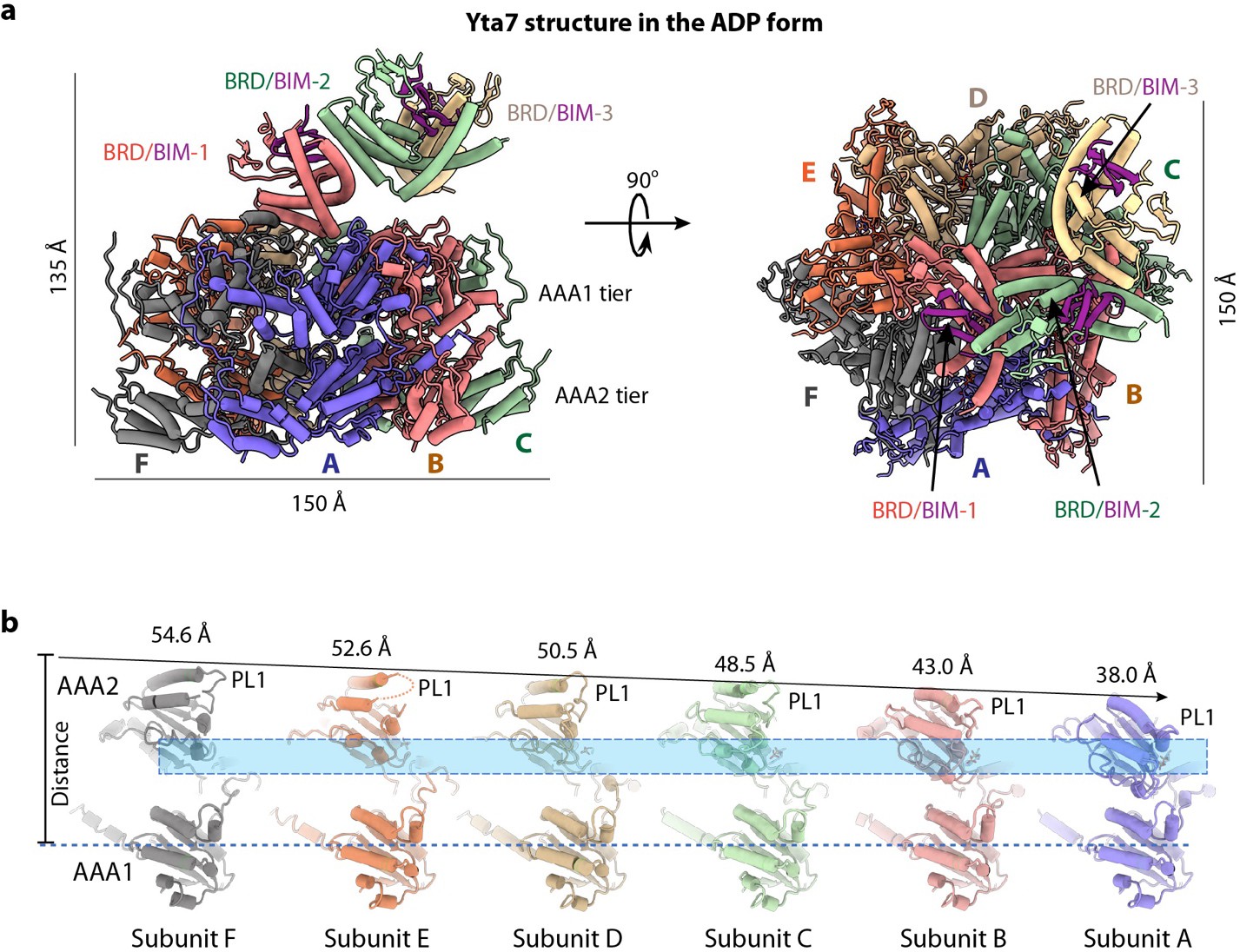


**Figure S7. Structure of the ADP-bound Yta7 hexamer. a**, A side and a top view of the ADP- bound Yta7 structure in cartoon. Subunits are individually colored. The three resolved BRD domains are in orange, dark green, and salmon, respectively, and their associated BIM motifs in magenta. Dimensions of the structure are shown in Å. **b,** The six Yta7 subunits are aligned with their respective AAA2 domains and shown individually in the same orientation. The distance between α2 helix of AAA1 and α3 helix of AAA2 in each subunit is labeled on top, revealing spiral arrangement of the AAA1 domains. The cyan rectangle marks the variably configured linkers between AAA1 and AAA2.


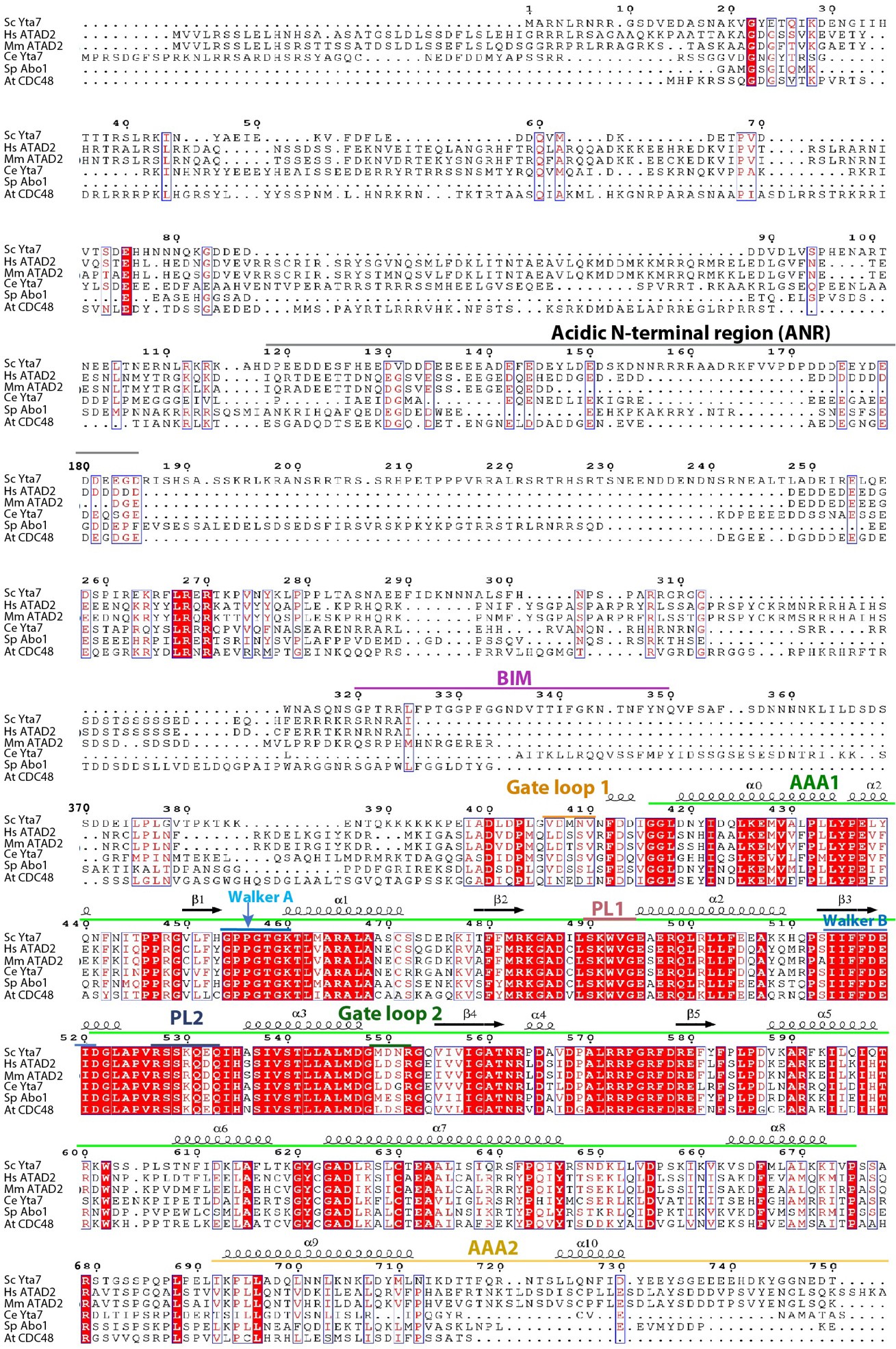


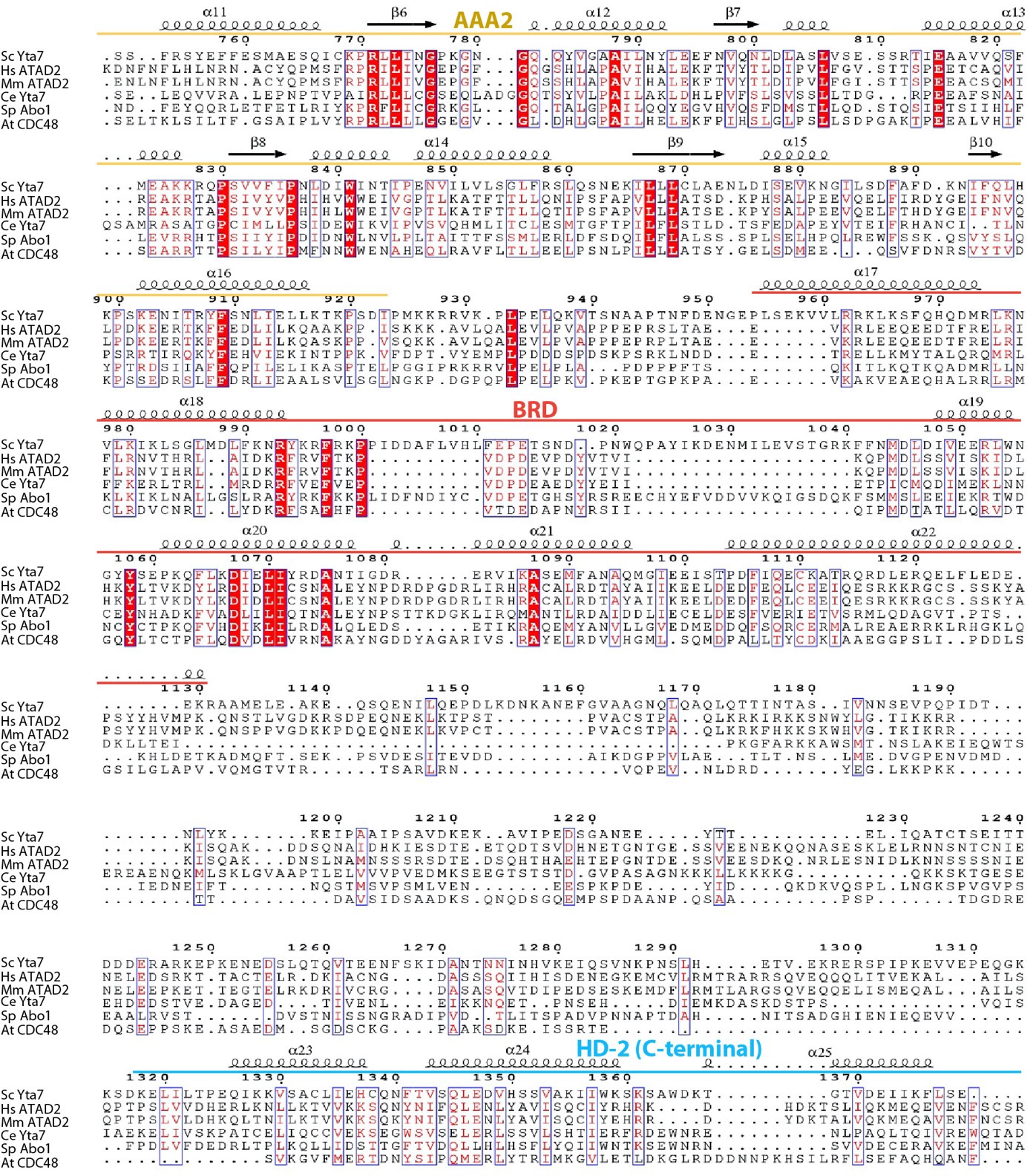


**Figure S8. Sequence alignment of six Yta7 homologues**. The amino acid sequences of *S. cerevisiae* Yta7, *H. sapiens* ATAD2, *M. musculus* ATAD2, *C. elegans* Yta7, *S. pombe* Abo1, and *A. thaliana* CDC48 were aligned in ClustalW and further analyzed by ESPript3.0. Major domains and key regions in Sc Yta7 are labeled above the sequence.


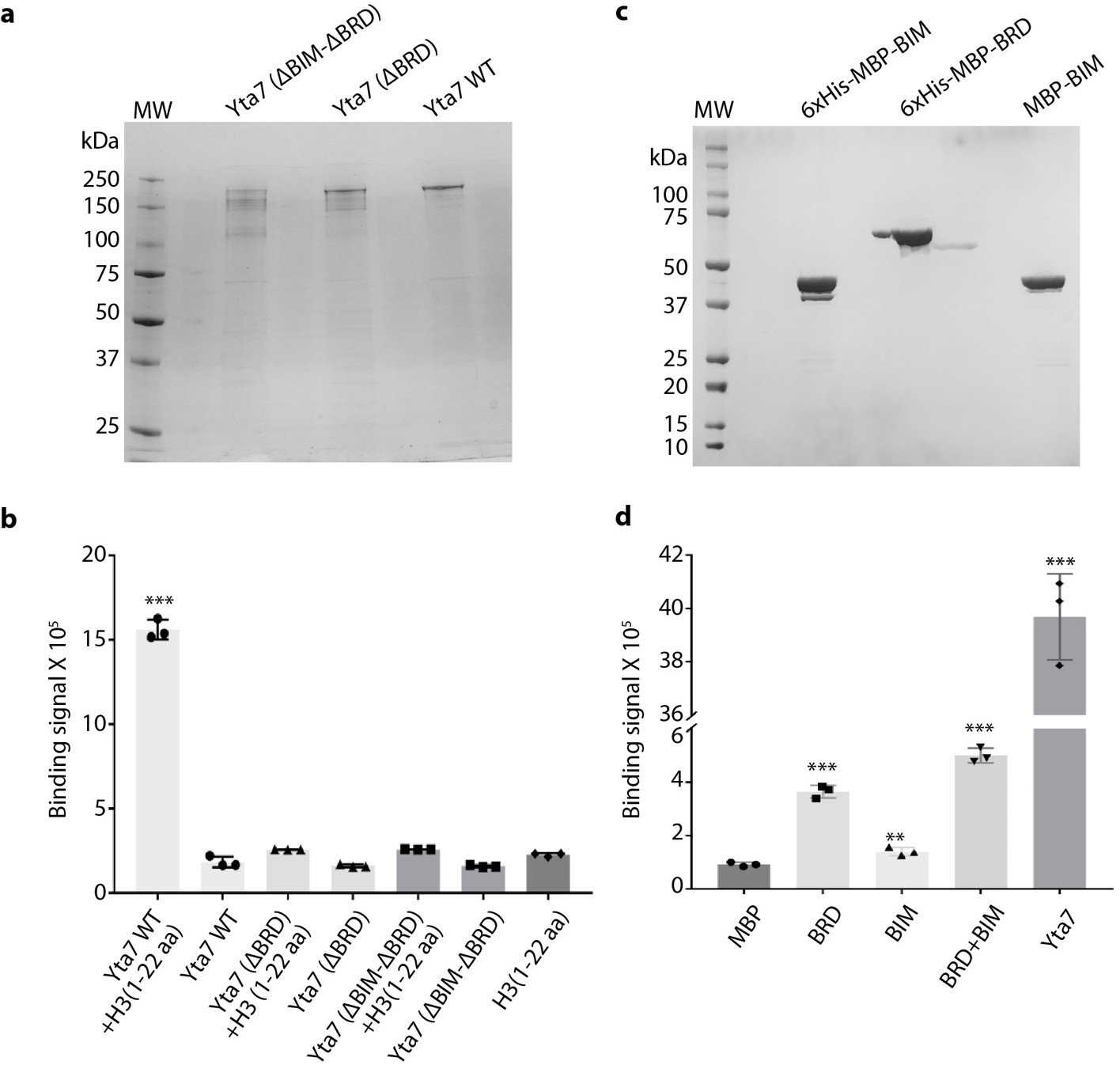


**Figure S9. Both BRD and BIM contribute to H3 binding. a**, SDS-PAGE gel of purified 10xHis-Yta7-(∆BRD) and 10xHis-Yta7-(∆BIM∆BRD) used in the AlphaScreen assay. Note that Yta7-(∆BIM) is not shown because this construct could not be expressed or purified. **b**,

Interaction of the H3 N-tail (1-22 aa, biotinylated) with Yta7 or Yta7 lacking BRD domain or lacking both BRD and BIM. Yta7 alone or Yta7 with BRD and BRM/BIM truncations was used as negative controls. (n = 3; error bars = SD). **c**, SDS-PAGE gel of purified 6xHis-MBP-BIM, 6xHis- MBP-BRD, and MBP-BIM used in the AlphaScreen assay. **d**, Interaction of the H3 N-tail (1-22 aa, biotinylated) either with BRD alone (with N-terminal His-tag and MBP fusion) or with NTR alone (with N-terminal His-tag and MBP fusion), or with the BrD–NTR complex (both are fused with MBP). The 6xHis-MBP served as a negative control (n = 3; error bars = SD). *** in panels b and d denote significant difference from controls (P ≤ 0.001, one-tailed t-test), ** (P ≤ 0.01, one- tailed t-test).


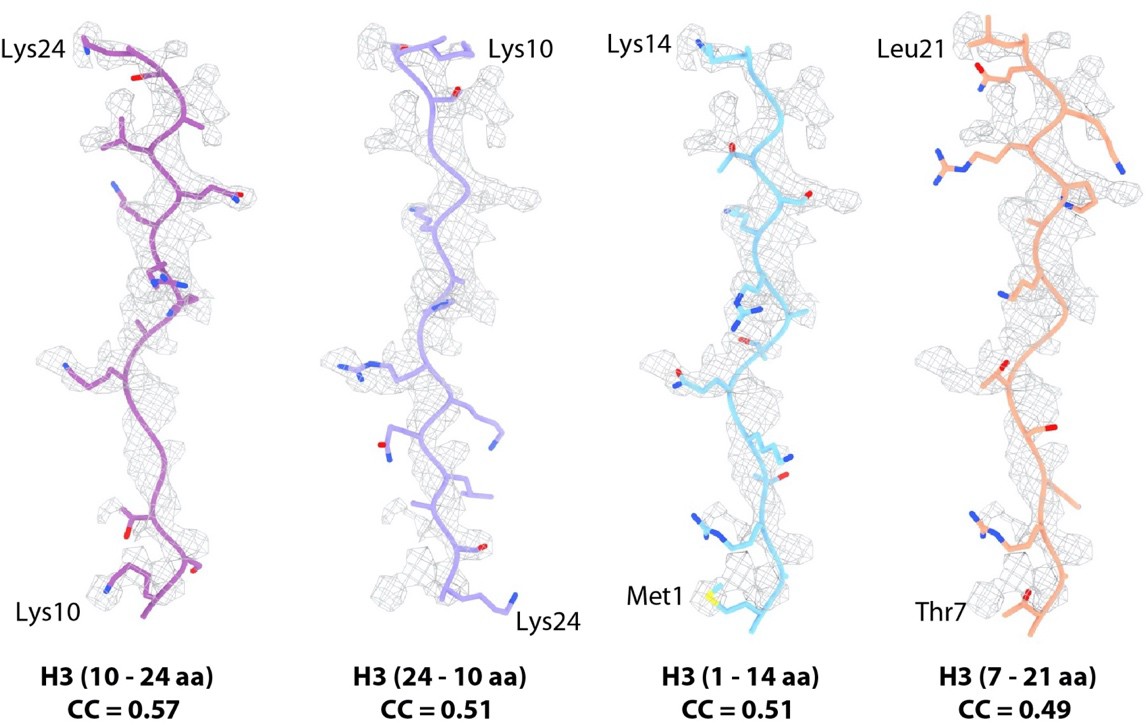


**Figure S10. Comparison of four alternative H3 peptide atomic modeling of the substrate EM density in the Yta7 ATPγS state II at 3.0 Å resolution.** In each panel, the peptide density is shown in gray mesh and the four models are shown in different colors with their respective N- and C-terminal residues labeled to indicate orientation. The model-to-map cross correlation peak coefficient is listed below each panel. The first model of H3 (10 - 24 aa) has the highest coefficient and apparently fits the EM density best.


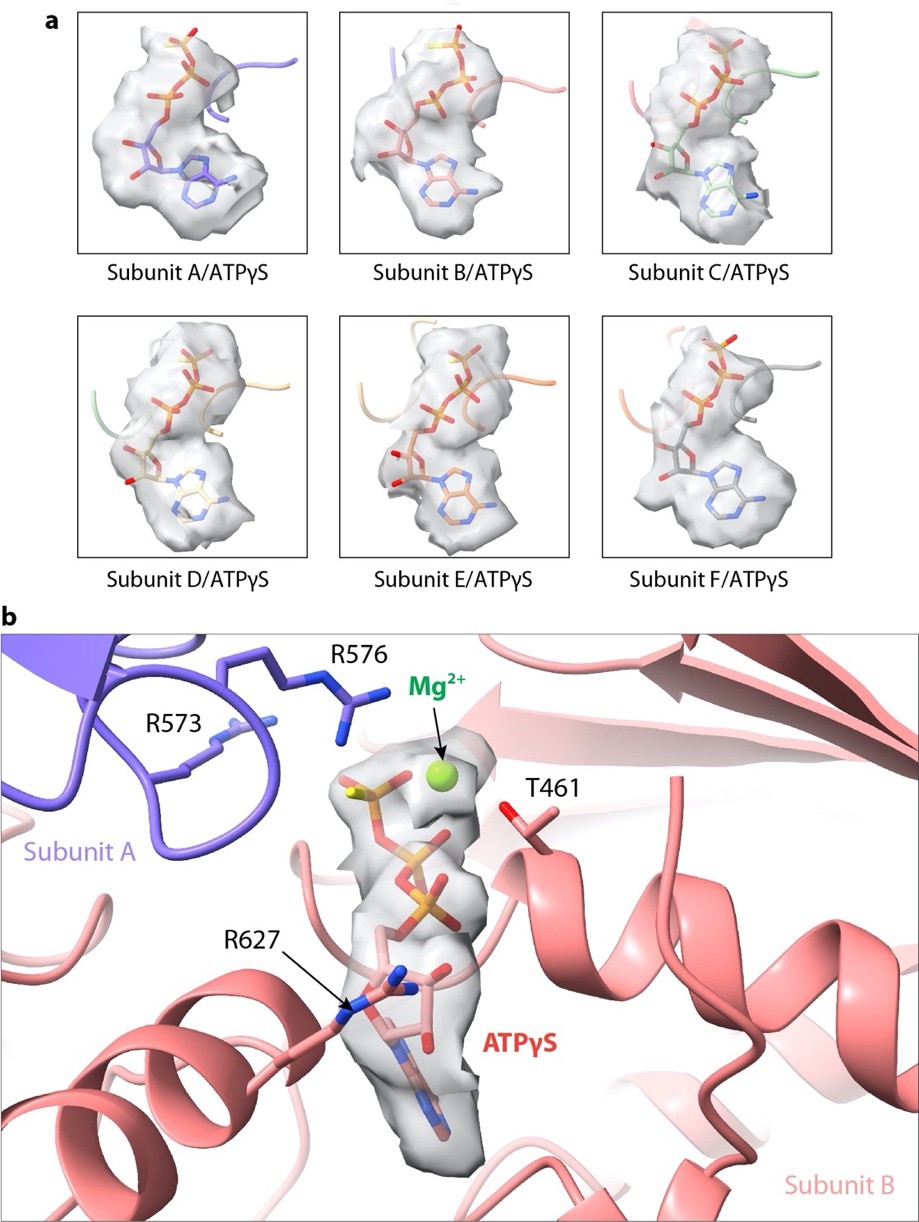


**Figure S11. Observed nucleotides in the AAA1 domains of Yta7 in the ATPγS bound states I and II. a,** In the ATPγS bound Yta7 state I structure, all six nucleotide-binding sites are occupied by ATPγS. ATPγS in stick view is superimposed in the transparent gray surface view of the EM density. **b,** In the ATPγS bound Yta7 structure state II, subunit B binds to ATPγS, but the coordinating Mg^2+^ has a weaker EM density that can be visualized only at a lower display threshold. ATPγS in stick view is superimposed in the transparent gray surface view of the EM density. The modeled Mg^2+^ ion is shown as a green sphere that fits well in the weak EM density.


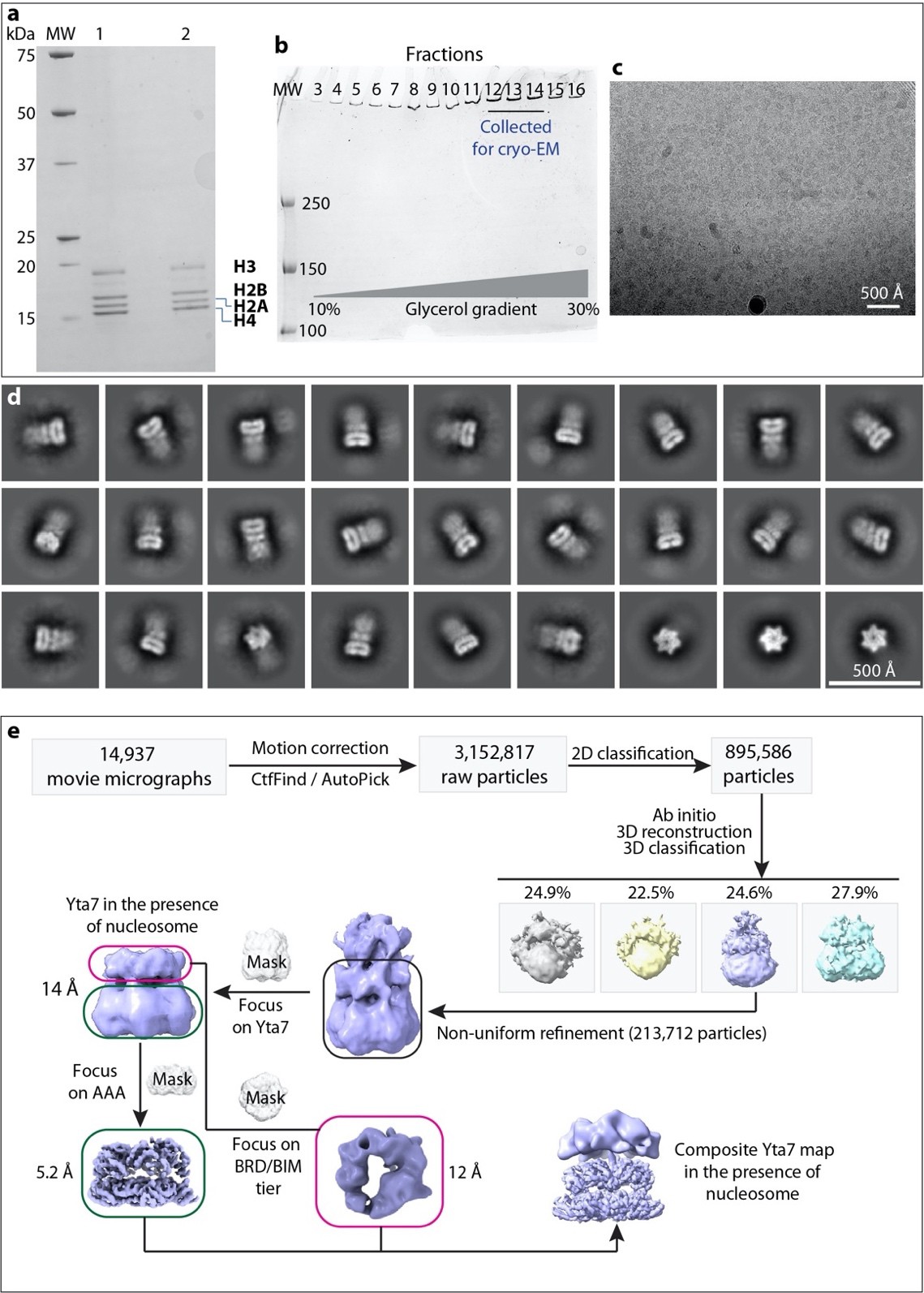


**Figure S12. Cryo-EM analysis of the Yta7-nucleosome complex. a,** SDS-PAGE gel of purified yeast histone octamer. **b**, SDS-PAGE analysis of the glycerol gradient fractions of the crosslinked Yta7–nucleosome complex. **c,** A typical raw micrograph of the Yta7–nucleosome complex bound to ATPγS. A total of 14,937 micrographs were recorded. **d,** Selected 2D class averages. The fuzzy density associated with the ordered Yta7 density is from the bound nucleosome. **e**, Workflow of image processing in cryoSPARC to derive the composite EM map of Yta7 in the presence of nucleosome.

**Supplementary References**

1.Chen S., Bell S.P., CDK prevents Mcm2–7 helicase loading by inhibiting Cdt1 interaction with Orc6 Genes Dev. 2011 363-372

2. Barad B.A., Echols N., Wang R.Y.-R., Cheng Y., Frank D., Adams P.D., EMRinger: side chain–directed model and map validation for 3D cryo-electron microscopy Nat. Met. 2015 943-946

3. Prisant M.G., Williams C.J., Chen V.B., Richardson J.S., Richardson D.C., New tools in MolProbity validation: CaBLAM for CryoEM backbone, UnDowser to rethink “waters,” and NGL viewer to recapture online 3D graphics Protein Sci. 2020 315-329
